# Antagonistic Coevolution Drives Whack-a-Mole Sensitivity in Gene Regulatory Networks

**DOI:** 10.1371/journal.pcbi.1004432

**Published:** 2015-10-09

**Authors:** Jeewoen Shin, Thomas MacCarthy

**Affiliations:** 1 Department of Applied Mathematics and Statistics, Stony Brook University, Stony Brook, New York, United States of America; 2 Laufer Center for Physical and Quantitative Biology, Stony Brook University, Stony Brook, New York, United States of America; Pennsylvania State University, UNITED STATES

## Abstract

Robustness, defined as tolerance to perturbations such as mutations and environmental fluctuations, is pervasive in biological systems. However, robustness often coexists with its counterpart, evolvability—the ability of perturbations to generate new phenotypes. Previous models of gene regulatory network evolution have shown that robustness evolves under stabilizing selection, but it is unclear how robustness and evolvability will emerge in common coevolutionary scenarios. We consider a two-species model of coevolution involving one host and one parasite population. By using two interacting species, key model parameters that determine the fitness landscapes become emergent properties of the model, avoiding the need to impose these parameters externally. In our study, parasites are modeled on species such as cuckoos where mimicry of the host phenotype confers high fitness to the parasite but lower fitness to the host. Here, frequent phenotype changes are favored as each population continually adapts to the other population. Sensitivity evolves at the network level such that point mutations can induce large phenotype changes. Crucially, the sensitive points of the network are broadly distributed throughout the network and continually relocate. Each time sensitive points in the network are mutated, new ones appear to take their place. We have therefore named this phenomenon “whack-a-mole” sensitivity, after a popular fun park game. We predict that this type of sensitivity will evolve under conditions of strong directional selection, an observation that helps interpret existing experimental evidence, for example, during the emergence of bacterial antibiotic resistance.

## Author Summary

Robustness, defined as tolerance to perturbations such as mutations and environmental fluctuations, is pervasive in biology. Previous models of gene regulatory networks have shown that robustness can evolve when the phenotype is under evolutionary pressure to remain constant (stabilizing selection). But in more realistic scenarios such as coevolution, it may be advantageous to evolve sensitivity, i.e. for some mutations to change the phenotype. We developed a two-population (host-parasite) model to investigate how robustness and sensitivity become distributed within a network under antagonistic coevolution. We found that sensitivity follows a pattern, similar to that of the game “whack-a-mole,” in which sensitive sites mutate, thus becoming insensitive, but new sensitive sites emerge to take their place.

## Introduction

Robustness, defined as tolerance to perturbations such as mutations and environmental fluctuations, is pervasive in biological systems [1, 2]. Early computational models of evolution aimed at understanding the relationship between gene-network evolution and behavior (gene expression dynamics) [3–5]. These studies found that, although a large number of different networks (genotypes) have the same gene expression dynamics (phenotype), they can usually be connected to one another via minimal changes (e.g. creation or deletion of single *cis*-regulatory interactions) that might easily occur during evolution via mutation. This capacity for neutral evolution can facilitate the evolution of robustness since it allows a population to migrate towards more robust genotypes without altering the phenotype [6]. Numerous theoretical studies have shown that robustness will evolve in particular when the phenotype is under evolutionary pressure to remain constant (stabilizing selection). Experimental results are consistent with this notion. Gene networks in *E*. *coli*, for example, have been shown to be robust specifically to regulatory rewiring [7]. Similar experiments on metabolic networks, also in *E*. *coli*, have shown network robustness with respect to both gene knockouts and network rewiring [8–10].

Many recent studies have shown that ecological interactions both within and between species, and particularly coevolutionary interactions, drive evolutionary changes on a far more rapid timescale than previously estimated [11–13]. Here we use network modeling to understand how coevolutionary selection, rather than stabilizing selection, evolves network structure and function and how coevolution determines evolutionary properties such as robustness and evolvability [11]. We focus on a simple case of antagonistic coevolution between two populations, specifically a parasite population that uses mimicry of a complex phenotype as its survival strategy, as well as its host population. There are many documented cases of such interactions. A well-studied example is brood parasitism of cuckoos on their avian hosts. For instance, cuckoo finches (*Anomalospiza imberbis*) deposit their eggs in the nest of their host, the African tawny-flanked Prinia (*Prinia subflava*). By mimicking the eggshell morphology of their hosts, the cuckoos trick their hosts into brooding these eggs. An evolutionary arms race between cuckoos and their host species drives continued variation in eggshell morphology in both species [14, 15]. In another example, coevolution of complex chemical signals occurs between *Maculinea alcon*, a parasitic butterfly species and their host, *Myrmica* ants [16]. *M*. *alcon* larvae emit a pattern of surface chemicals very similar to those of the ant larvae, leading the ants to adopt and feed the butterfly larvae as their own. An evolutionary arms race has arisen between these two species such that the ants evolve changes in their larval surface chemicals to discriminate their own larvae from those of the parasite whereas the parasite is continuously evolving to again produce a similar pattern.

It has previously been suggested that evolvability—the capacity for generating new phenotypes—can be facilitated by robustness, a somewhat counter-intuitive idea since evolvability and robustness would superficially appear to be opposite concepts. However, mutations will tend to accumulate in populations with high robustness, leading to greater genetic variation, which in turn may facilitate access to new phenotypes [1, 6]. Phenotypic variation might be accessible during episodes of directional selection or particular conditions such as environmental stress [1, 17–20]. Thus, under this model, periods of stabilizing selection allow genetic variation to accumulate, which is then eliminated by periodic selective sweeps and the cycle begins again with a new period of stabilizing selection. At the same time, the importance of this model remains unclear since few studies of network evolution have gone beyond stabilizing selection to investigate more realistic selection regimes [21]. Here we analyze host-parasite coevolution and find an entirely different strategy arises in which networks evolve a capacity for evolvability together with robustness against mutations. Here, evolvability in the network facilitates coevolutionary adaptation and is distributed throughout the network.

Previous studies have also shown there is a relationship between evolvability and modularity in networks. A strategy of using two target phenotypes presented, for example, in alternating succession has been used because it can select for distinct network modules, each of which is capable of generating one of the target phenotypes [22, 23]. One such study by Kashtan and Alon [22] used feed-forward logic networks and found that modularity evolved together with a fixed “evolvability node” which controlled the switch between two modules when mutated, thus switching phenotypes. Subsequent analyses showed evidence for modularity in other contexts including neural and metabolic networks [22, 24–26]. An alternative to a fixed “evolvability node” may be to have evolvability distributed throughout the network, allowing phenotype changes to occur in many different ways. Both types of evolvability are observed in nature [27]. Examples of fixed evolvability nodes include the Drosophila *shavenbaby* locus which predominantly controls trichome patterning [27], *Pitx1* which determines the pelvic spine phenotype in stickleback fish [28] and *optix* which controls rapidly evolving wing patterns in *Heliconius* butterflies [29]. Examples of distributed evolvability have been reported in bacterial and virus species including in *Helicobacter pylori* where a broad spectrum of genetic variations explains adaptation to its human host [30], in the pathogen *Pseudomonas aeroginosa* where antibiotic resistance evolves via several different mechanisms [31] and similarly in *E*. *coli* adaptation to low glucose environments [32]. Although the examples above illustrate the two extremes of what is likely a continuum between fixed and distributed evolvability, here we investigate a more general question—what conditions might favor the evolution of fixed vs distributed evolvability?

## Results

### Host-Parasite Coevolution Model

**Fig 1:**
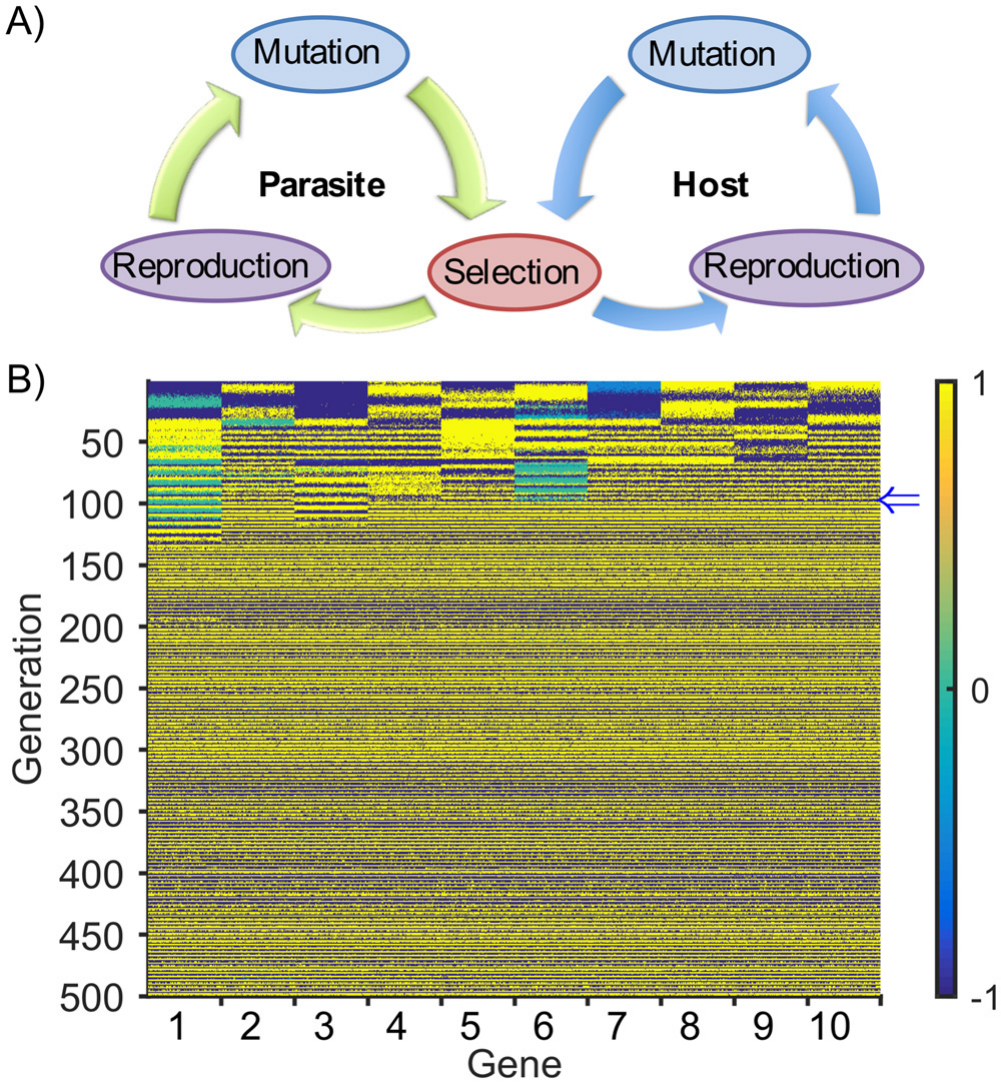
Host-parasite model and alternating phenotype dynamics. A) Schematic overview of the host-parasite model. B) To compare host and parasite phenotypes, here in a typical simulation, gene expressions are rescaled from [0,1] to [−1,+1] so that for each gene the sign of their multiplied gene expression indicates whether their expressions are similar or different. Host and parasite phenotypes are compared, here in a typical simulation, by multiplying the expression of each gene, rescaled from [0,1] to [−1,+1], from one host and one parasite at each generation. In the horizontal direction, the leftmost block of columns represents the comparative expression (by multiplication of rescaled expressions) level of gene 1 for 200 host-parasite pairs (the pairings themselves are random). Similar gene expression between host and parasite is shown in yellow (parasite winning) and divergent expression in blue (host winning).

To study gene regulatory network evolution under antagonistic coevolution we defined a model with two interacting populations. The model is an extension of a widely used single-population model that assumes stabilizing selection [33, 34]. As in the previous model, each population functions at two broad levels: genotype-to-phenotype mapping and population dynamics (see Methods for details). For the genotype-phenotype mapping, the genotype is defined as a gene regulatory network of *N* genes represented by a *N*×*N* matrix, *W*, the entries *w*_*ij*_ of which represent the regulatory strength and sign of gene *j* on gene *i*(*N* = 10 was used for all results unless otherwise stated). The genotype is mapped to phenotype via gene expression dynamics. The gene expression levels at time *t* are represented by *S*(*t*), a length *N* vector *S*(*t*) = [*s*_1_,*s*_2_, …,*s*_*N*_] (0≤*s*_*i*_≤1,*i* = 1, …,*N*). The genotype *W* defines a dynamical system that is used to determine steady state gene expression levels for each gene, which correspond to the phenotype, $\hat{S}$. Both host and parasite populations have a fixed number of individuals *M*. Cycles of reproduction, mutation and selection proceed in parallel as shown schematically in Fig 1A. Reproduction (either sexual or asexual) and mutation largely follow previous models [35–37]. Genotype mutations allow for creation and deletion of regulatory interactions as well as quantitative changes [36]. The main difference with previous models is at the selection stage, where the host and parasite populations interact by mutually determining fitness in the other population. To represent antagonistic coevolution in our model we assume that a candidate parasite individual has higher fitness when its phenotype is similar to that of a randomly chosen host individual (a new random host is chosen for each parasite at every selection step and similarly for each host). Thus parasite fitness is defined as: $f\left({\hat{S}}_{P}\right)=e^{-\frac{D({\hat{S}}_{P},{\hat{S}}_{H})}{\alpha }}$, where $D\left(X,Y\right)=\frac{\sum_{i=1}^{N}{\left(x_{i},y_{i}\right)}^{2}}{N}$, ${\hat{S}}_{P}$ is the parasite phenotype, and ${\hat{S}}_{H}$ is the phenotype of a randomly selected host individual. On the other hand, we assume the host has higher fitness when its phenotype is different from that of the parasite and therefore host fitness is defined as: $f\left({\hat{S}}_{H}\right)=e^{-\frac{1-D\left({\hat{S}}_{H},{\hat{S}}_{P}\right)}{\alpha }}$ where ${\hat{S}}_{P}$ is the phenotype of a randomly selected parasite individual. *α* is a parameter representing selection pressure. The fitness functions are symmetric about *x* = 0.5 (S1 Fig) to avoid any bias in how selection is applied in host vs parasite. Although the initial phenotypes are random, this two-population approach allows the eventual target phenotypes to emerge from the model, in contrast to previous models where the target phenotypes are defined *a priori*. The fitness definitions used are analogous to the two examples of host-parasite evolutionary arms races described above (cuckoo finch and *M*. *alcon*) whereby similarity (and differences) in complex phenotypes are selected for: eggshell morphology in the case of the cuckoo finch or the pattern of larval surface chemicals in the case of *M*. *alcon*.

### Host and Parasite Populations Evolve Networks with Distributed Sensitivity and Robustness

Under sufficiently strong selection pressure (*α*) both host and parasite populations reach a stage where their phenotypes alternate between one phenotype $\hat{S}$ and an approximately “inverted” version of the same phenotype, i.e. $1-\hat{S}=[1-s_{1},\;1-s_{2},\;\;\ldots ,\;1-s_{N}]$. At a given generation, if the host population phenotype is ${\hat{S}}_{H}$ and that of the parasite is ${\hat{S}}_{P}=1-{\hat{S}}_{H}$, then the host will have high fitness and the parasite will have low fitness. However, if at a later generation the parasite population is able to “invert” its phenotype ${\hat{S}}_{P}\to 1-{\hat{S}}_{P}(={\hat{S}}_{H})$ and the host population maintains its phenotype (${\hat{S}}_{H}$), then the parasite and the host phenotypes will become the same—the host will now have low fitness and the parasite will have high fitness. The parasite population will continue “winning” until the host population is able to invert its phenotype, and the cycle continues. Fig 1B and S2 Fig show this progression over time (vertical axis) for every gene expression level in every gene (horizontal axis) of every individual in a typical simulation (see Methods for parameter values used). Each cell in Fig 1B is colored blue when the expression level favors the host “winning” (i.e. when a host gene is on and the corresponding parasite gene is off and vice versa), and yellow if the parasite is “winning” (i.e. the host and parasite levels are the same). We see that by generation ~100 (blue arrow Fig 1B) both populations have converged to an alternating strategy as the rows alternate in color. Thus both host and parasite genotypes have become highly evolvable in response to phenotype changes in the opposite population. We are primarily interested in how these coevolutionary interactions between host and parasite populations affect gene regulatory network evolution and in particular how evolvability itself evolves within the networks. As expected, under weaker selection (approximately *α*>0.15, see S3A Fig) the alternating phenotype did not evolve, so we focused here on the stronger selection case.

We next sought to identify the mechanism underlying the phenotype inversion process, i.e. the evolution of evolvability. One possibility is that the alternating phenotype strategy would evolve in the form of a particular “evolvability hotspot” or interactions in the network, analogous to those identified previously by Kashtan *et al*. [22] in modular networks. A mutation in an “evolvability hotspot” would be highly likely to cause a phenotype inversion. An alternative scenario is one in which the capacity for phenotype inversion is highly distributed, and phenotype inversion can occur in many different places throughout the network, albeit with low probability. To assess these effects we implemented two measurements: first, a sensitivity score (*SS*) that estimates the overall probability that a mutation will cause a phenotype inversion (see Methods), and secondly a measure of how distributed the sensitivity is within the set of network interactions that can cause a phenotype inversion, as described below. In addition, to measure the effects of coevolution on the remaining parts of the network (that do not cause phenotype inversions) we also quantify mutational robustness in this subset of network interactions.

**Fig 2:**
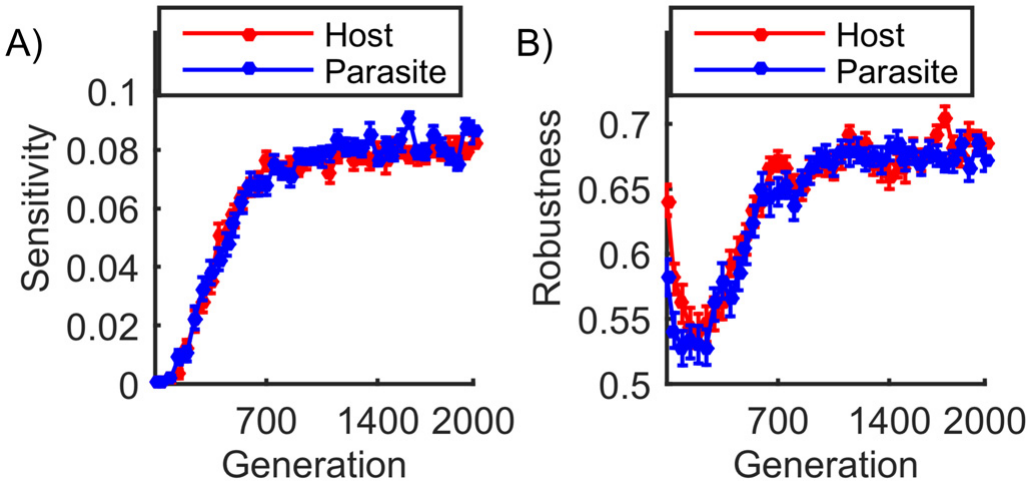
Emergence of sensitivity. A) As coevolution proceeds, the sensitivity score (*SS*) increases monotonically reaching a plateau in both host and parasite. B) Robustness in the remaining (non-sensitive) part of the network was defined as the fraction of mutations that leave the phenotype unchanged if we exclude phenotype inversions (see main text). Both plots show mean values for 100 simulations with the error bars indicating standard error of the mean (SEM).

Fig 2A shows the progression of the sensitivity score (*SS*) during a typical simulation. Here we see that at the beginning of the coevolutionary process, because host and parasite networks are random, both have a negligible number of sensitive interactions and the mean *SS* is close to zero. As antagonistic coevolution proceeds and both populations evolve towards the alternating phenotype strategy, they both acquire sensitive interactions and the mean *SS* increases, eventually reaching a plateau. For the set of parameters shown in this example (see Methods), *SS* reaches approximately 0.08. Although this qualitative behavior is observed across a large range of parameter values, there are quantitative differences. Thus, the steady state *SS* level is reduced, as expected, if selection pressure is lower (S3A Fig) and with smaller population sizes (S3C Fig) where random drift effects are greater. Also, networks with a greater density of connections can evolve sensitivity more easily (S3E Fig). Lastly, note that multiple simultaneous mutations can occur within a single genotype, particularly when the frequency of the single mutation is high, as is often the case when a population is undergoing a phenotype inversion (S4A and S4B Fig). Although such events occur at low frequency, we found that, in cases of double mutations at least one of the mutation positions had a high sensitivity score whereas the other usually had a sensitivity score that was either very low or zero (S4C Fig). Thus, a phenotype inversion is most often achieved with a single point mutation at a sensitive interaction, although occasional double mutations where at least one mutation is at a sensitive interaction can also cause a phenotype inversion.

The sensitivity score (*SS*) estimates the probability of causing a phenotype inversion. We found that the capacity for causing a phenotype inversion is distributed across a large number of sensitive network interactions, and we therefore sought to quantify how sensitivity was distributed throughout the network. Sensitivity might either be distributed fairly equally among these interactions, or unequally in the sense that particular interactions are likely to cause a phenotype inversion whereas others interactions do so only with low probability. To quantify the distribution of sensitivity we first chose the subset of network interactions, *w*_*ij*_, that exhibit sensitivity, where the subset is defined as those having a (interaction specific) sensitivity score *SS*_*ij*_>0 (see Methods). We compared the observed standard deviation (SD) of the *SS*_*ij*_ values to the SD of a null model that assumes the observed total sensitivity in this set of nodes is randomly distributed (see Methods). We consistently found that the null model has a comparable, and even slightly higher, variance of sensitivity within the sensitive interactions than the evolved networks (S5A Fig). Thus the levels of sensitivity are at least as similar amongst themselves than would be expected by chance given the observed total sensitivity in the network (see Methods).

Apart from causing a phenotype inversion, a mutation may either (*a*) leave the phenotype unchanged, which indicates robustness, or (*b*) cause the phenotype to change only partially which will usually be sub-optimal. As a measure of robustness, Fig 2B shows the fraction of mutations that leave the phenotype unchanged if we exclude phenotype inversions, i.e.(*a*)/((*a*)+(*b*)). These results show that robustness initially decreases but then increases, eventually reaching a level higher than that of the initial population.

Note that the initial host and parasite populations have random phenotypes, generally their phenotypes are not in either similar or inverted forms. In addition sensitivity does not exist before coevolution. Therefore during the initial phase, all host/parasite individuals are under evolutionary pressure to explore alternative phenotypes to counter the other (parasite/host) population, which is also in a similar situation. Partial phenotype changes will therefore be beneficial until both populations enter the process of phenotype inversion. This is why robustness decreases in the earliest stages of coevolution (Fig 2B). However, once the capacity for phenotype inversion has evolved, partial phenotype changes will not be beneficial especially under strong selection and there is selection pressure for mutations to either preserve or invert the phenotype. This is why robustness increases together with sensitivity, and why robustness eventually exceeds the initial (pre-selection) levels. Again, we found that the phenomenon of increased robustness is observable across a wide range of parameter values although the range is more limited under sexual reproduction than it is with asexual reproduction (S6 Fig). Generally though, robustness evolves in the parts of the network that are not causing phenotype inversion. Thus in the steady state, both robustness and evolvability coexist in the network under coevolutionary selection.

Although we observe that mutational robustness evolves under antagonistic coevolution, environmental robustness appears to coevolve to a much lesser extent. Previous studies have shown that even without direct selection for environmental robustness, mutational and environmental robustness will coevolve under stabilizing selection [38, 39]. Environmental robustness was evaluated via perturbations of the initial gene expression levels and then by measuring the phenotypic distance between the perturbed and unperturbed cases (see Methods). Given the trend for mutational robustness (Fig 2B), the overall pattern was similar to that expected (S7 Fig). However, the phenotypic distance increased to steady state levels that were well above those observed initially, indicating an overall reduction in environmental robustness. This was the case regardless of whether the perturbation rates were low or high relative to the mutation rate.

We have addressed the simple case of equal population sizes for host and parasite. This case is relevant to many real host-parasite interactions such as the example of the cuckoos and their avian hosts discussed above, where the populations appear to be relatively stable and of comparable size [40]. Clearly however, host and parasite populations will often differ in size. We therefore evaluated the case of host population size = 100 and parasite population size = 1000 (and vice-versa), finding only slight differences with the case of equal population sizes (S8 Fig vs. Fig 2). However, due to computational constraints we were unable to model much larger population sizes and we therefore leave a more thorough evaluation of unequal population sizes for future work.

### Sensitive Regulatory Interactions are Highly Labile throughout Evolution

**Fig 3:**
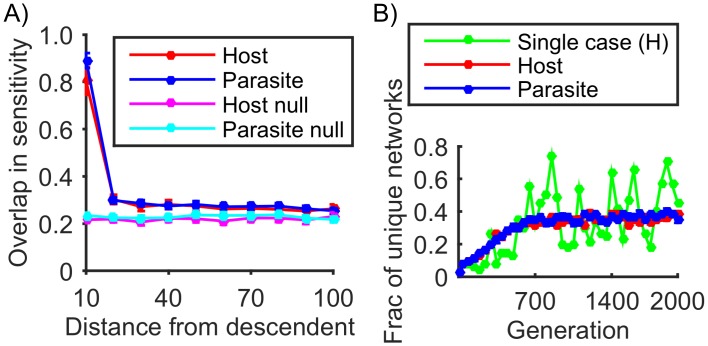
Lability of sensitive interactions and network diversity. A) Comparison of the sensitivity network interactions from a single individual in a population at generation 2000 with its ancestors using the Jaccard index to quantify the overlap in sensitivity (see Methods). (B) Time course of network diversity, defined as the number of distinct networks, simplified to sign (-1/0/+1) form, and expressed as a fraction of the population (green line: host population of single simulation). Apart from the green line in (B), both plots show mean values for 100 simulations with the error bars indicating SEM.

We next investigated whether sensitivity is preserved at particular points in the network or whether it changes over time. As described above for the case of modular networks, sensitivity will often evolve to be focused on “hotspots” that control distinct phenotypes and which do not change over time [22, 24]. To assess the changes in the sensitive interactions over time we used asexual reproduction. Under asexual reproduction, tracing the ancestral lineage is straightforward because there is a single parent for each individual, and after *G* generations each individual in the population needs *G* ancestral genotypes to store its genetic history. In contrast, under sexual reproduction each individual needs at most 2^1^+2^2^+⋅⋅⋅+2^*G*^ ancestral genotypes, which rapidly becomes unwieldy. We consider the set of sensitive points of the network (i.e. those interactions *w*_*ij*_ with sensitivity score *SS*_*ij*_>0 that may cause a phenotype inversion) and how this set changes over time. We selected networks at a particular steady state generation and compared these to ancestral networks at various evolutionary distances. The comparison was done by measuring the similarity, in terms of sensitivity, between the ancestral and derived networks using the Jaccard index (see Methods) as shown in Fig 3A. Given that the phenotype is constantly changing, to ensure a valid comparison we only compared with ancestral networks having the same phenotype. As shown in Fig 3A, the overlap in sensitivity remains high only for a short time period, before dropping almost to levels that would be expected by chance (null model Fig 3A—also see Methods). However, at steady state the sensitivity remains stable, as do the total number of sensitive interactions (generations ~1000 onwards, Fig 2A and S5B Fig). Thus, sensitive interactions are highly labile and on average, each time a sensitive interaction is eliminated by mutation, a new one emerges to take its place. Colloquially this property is known as “whack-a-mole”, named after the fun park game, and we therefore refer to this phenomenon as whack-a-mole sensitivity.

**Fig 4:**
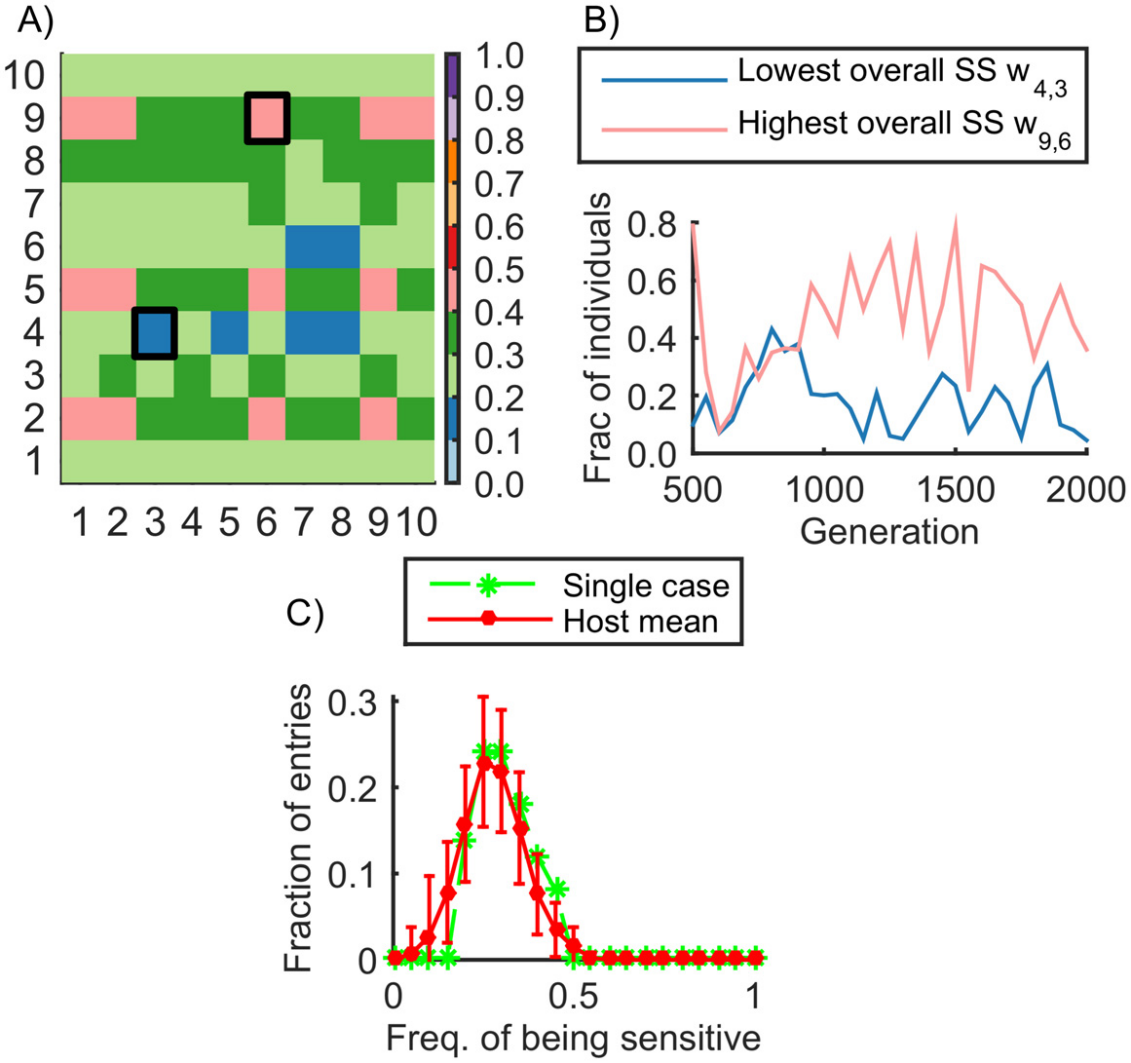
Distribution of sensitivity throughout the network. A) Frequency of being a sensitive interaction in the *N*×*N* matrix of interactions (with *N* = 10) in a typical simulation. From generation 500 onwards we identified the sensitive gene interactions *w*_*ij*_ (*SS*_*ij*_>0), then measured the frequency for each *w*_*ij*_ being sensitive within the population, at intervals of 50 generations. We sum the frequencies over time and normalize to the interval [0,1] as indicated by the colors. Generally there are no interactions that appear to dominate within each population over many generations. B) Detailed progression of sensitivity over time for two particular interactions in (A). These interactions had the lowest (blue) and highest (pink) overall sensitivity, as indicated by the black squares in (A). C) Distribution of the frequency of being sensitive in all *N*×*N* interactions for all host individuals (green dashed line: the host population of (A), red solid line: mean of 100 simulations). Since distributions are mostly right-skewed there are no interactions that dominate in terms of sensitivity. Error bars indicate one SD.

Even though sensitive interactions are labile and are constantly being relocated, we thought there might be a specific subset of interactions with consistently high sensitivity. Alternatively there might be no persistence in the sensitive interactions or any such interactions would be rapidly lost. Consistent with the latter scenario we found there are no interactions with a significantly high frequency of being a persistent sensitive interaction within a population and throughout a simulation, as shown in Fig 4. Fig 4A shows, for a typical simulation, the frequency at which each interaction *w*_*ij*_ was sensitive over a period of 1500 generations while sensitivity and robustness were at steady state levels. Fig 4B shows the change in sensitivity over time for two particular interactions in Fig 4A (those that had the highest and lowest overall sensitivity respectively). Fig 4C shows the same data in histogram form (green curve) together with the mean value for many simulations (red curve). Even though there appears to be no preference for particular positions within the matrix, we tested whether there was a higher-level preference for particular rows of the interaction matrix *W*, which represent the *cis*-regulatory elements for each gene. For this, we considered the total sensitivity score for each row (*i*), *SS*_*i*_, and in particular, tracked the row *i*_*max*_ for which the value of *SS*_*i*_ is maximal within each individual (S9A Fig). We found that rarely does a particular *i*_*max*_ dominate both the population and throughout generations (S9B Fig). We repeated this analysis for columns, which represent gene outputs regulating genes, finding similar results. Thus, there does not appear to be any predilection for sensitivity to be associated with particular genes.

### Antagonistic Coevolution Drives High Levels of Diversity

As explained in the introduction, another way by which phenotypic innovation has been proposed to occur is through increased genetic variation, which is promoted by robustness. However, if a sensitivity mechanism has evolved to generate the appropriate phenotype changes, it does not, in principle, require high levels of genetic variation to function. To investigate the observed levels of genetic variation in the population that has evolved sensitivity we used a measure that simplifies each network using the sign of each matrix entry sgn(*w*_*ij*_), then counts the number of distinct (simplified) networks, expressed as a fraction of the population. Fig 3B shows how this diversity measure increases over time. Taking a typical host case (green curve) as an example we found that in the final population there were 91 distinct networks, which expressed as a fraction of the total population, leads to a diversity measure of 91/200 = 0.45. The average trend (red and blue curves) shows diversity increasing over time, eventually reaching a plateau. This diversity is a consequence of the beneficial mutations (occurring at sensitive interactions) being broadly distributed throughout the network, thus making multiple evolutionary pathways available. Taking this analysis further, we used the same diversity metric to measure the level of variation generated by stabilizing selection (see generations 1–500 in S10 Fig) and found that the level of diversity was consistently below that observed under antagonistic coevolution. Although the comparison needs to be interpreted cautiously given that stabilizing and coevolutionary selection are quite different, we include it here to emphasize the high degree of diversity observed under coevolution. This high diversity occurs despite there being, in principle, no requirement for it.

Lastly, we assessed the impact of having an initial phase of stabilizing selection that allows each population to evolve robustness and accumulate genetic variation independently before the coevolutionary process begins. As shown in S10 Fig (generation 500 onwards), genetic variation increases under the initial stabilizing selection phase (previous model of [34] was used for this), reaching a plateau by generation ~100. Once coevolution begins, at generation 500, genetic diversity is reduced as both populations pass through a bottleneck but then increases eventually exceeding the level achieved under stabilizing selection. However, the dynamics are not significantly different from those observed without the initial phase of stabilizing selection, and therefore the initial phase appears not to offer any advantage.

### Innovation Arising from Sensitivity Does Not Require Modularity

As mentioned in the Introduction, previous studies [22, 24, 26, 38] have investigated the conditions leading to increased modularity in a network using multiple target phenotypes. In each case, the target phenotypes contained a combination of features such that a modular network evolves. In particular, the study by Espinosa-Soto and Wagner [23] used two target phenotypes that only overlapped partially, leading to increased modularity (S11A Fig, red curve). Applying the same modularity measure to our own simulations (see Methods), we found that increased modularity did not evolve for any combination of parameters. We thought this might be because in our model, the entire phenotype alternates, in contrast to only part of the phenotype in the Espinosa-Soto model. However, a variant of our model in which only half the phenotype genes participate in host-parasite fitness also did not evolve modularity (S11A Fig, green curve). The other key differences between the two models are how gene interactions and expression levels are represented (real vs discrete), the method of presentation of target phenotypes (sequentially alternating vs simultaneous) and the type of perturbation (mutational vs environmental), as summarized in S11B Fig. We therefore tested variant models that contained mixtures of features from either of the models but were unable to find increased modularity for any of the variant models (S11A Fig). These results suggest that modularity will evolve only under the very specific conditions. Biologically, the most important of these conditions is perhaps the nature of the perturbations, which can broadly be interpreted as growth-related or developmental for our model vs physiological or environmental in the case of the Espinosa-Soto model.

Another relevant study, by Kashtan and Alon [22] also found that modularity evolved in the network together with persistent sensitive nodes. This was achieved using alternating target outputs, so-called Modularly Varying Goals (MVGs), which were defined as pairs of logical functions containing different combinations of sub-goals. For example, the authors defined two functions (of 4 inputs, X, Y, Z and W) as G1: (X XOR Y) OR (Z XOR W), G2: (X XOR Y) AND (Z XOR W). Although the model used was based on logical circuits and therefore quite different to the one we have used here, we evaluated whether using this particular pair (G1, G2) of MVGs would also result in long-term sensitive nodes. We implemented this using a single population model with networks having 4 designated input genes and 6 interacting regulatory genes, one of which is considered the output. Since there are 2^4^ = 16 possible inputs, fitness was defined as the fraction of correct input-output mappings. We evolved the population using alternating targets (G1, G2, G1, …) for 50 generations per target. For a population evolved under one target (e.g., G1), we assessed sensitivity, and in particular we considered any mutated network as sensitive if it matched the alternate target (e.g., G2) in more than 12 out of 16 input-output pairs (i.e., a fraction of 0.75). The threshold was set to 0.75 because we did not observe the average fitness exceeding this level for either target (S12A Fig). As shown in the S12B Fig, we do observe that a subset of persistent sensitive network nodes evolves; this is the subset of nodes with frequency of sensitivity equal to 1. However, in contrast to the previous study we did not observe increased modularity over time (S12C Fig), presumably because most sensitive interactions are not persistent, but highly transient, with frequencies of sensitivity between zero and one (S12B Fig). To further confirm that it is indeed the MVGs that facilitate the appearance of the subset of persistent sensitive nodes, we checked two further scenarios using the single population model. Firstly, we used two alternating targets in which half of the target genes (*N*/2) are kept the same as the founder phenotype and the other half are inverted every 50 generations. This model is similar to that of Espinosa-Soto described above, except that the targets alternate in time, rather than being selected for simultaneously. In the second case, we simply alternated between the founder phenotype and its inverted form, again every 50 generations. In neither of these cases did we observe the emergence of persistent sensitive nodes (S13 Fig) as we observed with the MVGs.

A second important difference between our approach and that of Kashtan and Alon lies in the mutation model. The Kashtan and Alon study used only topology changes, whereas our approach allows for both quantitative interaction modifications and topology changes. To investigate this difference further, we used our model to evaluate differences in the contribution of weight modifications vs topology changes. We found that increasing the relative importance of topology changes (by increasing the parameters for addition, *ρ*, and deletion, *ϕ*) did not qualitatively change our results and in particular, did not create persistent sensitive nodes in the network (S14A and S14B Fig). A reduction in the relative use of topology changes (by reducing *ρ* and *ϕ*) also did not change results qualitatively (S14C and S14D Fig). In conclusion, these analyses suggest that MVGs explain the major difference in outcomes, namely persistent evolvability nodes in the Kashtan and Alon model compared to distributed and labile evolvability nodes in our host-parasite coevolution model.

## Discussion

Robustness—defined as tolerance to perturbations such as mutations and environmental fluctuations—is a pervasive feature of biological systems [17, 38]. Previous models of gene regulatory networks have shown that mutational robustness evolves under conditions of stabilizing selection [34, 38]. However, under more realistic scenarios, such as coevolution, evolvability may be advantageous. It is unclear though, how sensitivity and robustness will evolve and in particular how they will become distributed throughout a regulatory network. To investigate this we developed a two-population (host-parasite) model of antagonistic coevolution. Although previous studies [22–24] had investigated the evolution of sensitivity in networks under fluctuating environmental conditions, a key novelty of our model is that the fitness landscapes are emergent properties of the inter-population interactions. This approach avoids the need to impose a changing environmental regime externally. Furthermore, the pace of evolution is dictated largely by the model’s ability to adapt. Self-contained models such as these represent a step towards open-ended evolutionary models that will be critical in the longer term to understanding how biological complexity evolves.

We found that sensitivity increases after the initiation of coevolution and becomes highly distributed throughout the network. At the same time, the remaining (non-sensitive) parts of the network evolve to become robust. Interestingly, genetic diversity evolves to be higher under antagonistic coevolution than under stabilizing selection. There are two obvious sources of diversity in this case. Firstly, in the non-sensitive parts of the network, robustness facilitates the accumulation of genetic variation via a well-understood mechanism [41]. Secondly, because sensitivity is distributed across the network, there are many different ways in which mutations cause phenotype inversions, contributing to diversity particularly after several rounds of selection. If sensitivity were not distributed, but were focused on a particular “evolvability hotspot”, genetic diversity could in principle be far lower.

We found that robustness evolves in the parts of the network that are not involved in phenotype inversion. Interestingly, this robustness evolves more easily under asexual reproduction (Fig 2B) than sexual reproduction (S6B Fig). Generally speaking we found that under sexual reproduction, a combination of higher network density, stronger selection pressure and/or larger population size was required in order to attain levels of robustness comparable to the asexual case, suggesting that recombination load is having nontrivial effects under sexual reproduction. In support of this, theoretical population genetic studies investigating the evolution of recombination [42, 43] have shown that asexual reproduction will be favored over sexual reproduction under antagonistic coevolution when the two modes are allowed to compete. At the same time, a previous study using a similar network model to ours [44], but having a single population under conditions of stabilizing selection, demonstrated that recombination load evolves to minimal levels under stabilizing selection. Thus it would appear that recombination load evolves to be higher under antagonistic coevolution than under stabilizing selection.

We found that coevolutionary selection drives networks to evolve labile sensitivity such that evolvability and robustness are continuously redistributed throughout the network. Sensitive points within the network cause a phenotype inversion when mutated (from $\hat{S}\to 1-\hat{S}$), but the mutation by definition also changes the genotype, in particular by causing a change *w*_*ij*_→*w*_*ij*_'. Assuming the lineage continues through another phenotype inversion (from $1-\hat{S}\to \hat{S}$) and *w*_*ij*_' is not mutated again, then *w*_*ij*_' will most likely no longer be sensitive. However, each time a sensitive point in the network is “used up”, a new sensitive point emerges elsewhere, thus maintaining overall sensitivity at approximately constant levels. We refer to this process as whack-a-mole sensitivity, named after a fun park game in which targets are removed from one place only to reappear elsewhere. A comparable whack-a-mole process also appears to occur with meiotic recombination hotspots in mammals [45, 46]. During meiosis, recombination breakpoints are frequently initiated at DNA motif hotspots recognized by the PRDM9 protein. However, DNA repair mechanisms cause hotspots to be preferentially lost in the gametes of heterozygote (hotspot/non-hotspot) individuals and the net effect is for recombination hotspots to be lost over time. However, by means that are still not well understood, the overall number of hotspots (in humans for example) remains approximately constant while the positions of recombination hotspots are transient and vary within humans [47, 48], suggesting there must be a mechanism for generating new hotspots to replace those that have been lost, i.e., a whack-a-mole process. Broad distributions of mutations have been observed in antibiotic resistance, for example in bacteria which produce extended-spectrum beta-lactamase (ESBL) enzymes [49, 50]. In this case, many distinct point mutations occurring in ESBL genes such as TEM–1 and SHV–1 transform the active site of the enzyme. More than 330 ESBL variants including TEM- and SHV- type variants have been reported [50]. Whack-a-mole sensitivity may explain these rapidly expanding mutations in genes encoding ESBLs, thus helping to predict the evolution of resistance.

In our model the ongoing phenotypic inversions are dependent on successive mutations that accumulate across many different loci. Because we found that sensitive nodes are labile (whack-a-mole sensitivity), this means that over time similar mutations at a particular gene regulatory interaction might have distinct phenotypic effects. For example, a phenotype inversion (*S*→1−*S*) might be caused by a mutation at a particular sensitive site *w*_*ij*_. The original phenotype *S* might then be restored by a reverse mutation at the same site. However, because sensitivity is distributed across the network, the restored phenotype *S* is more likely to arise through a mutation at some other site different than *w*_*ij*_. Indeed several generations may pass before this reverse mutation occurs and by that time other mutations may have accumulated in the network. In this new genetic background the “reverse” mutation may no longer have the same effect. This is a clear example of serial epistasis—the dependency of mutational effect on the genetic background established by previous mutations [51]. A widely-cited study of serial epistasis in a natural population involves the evolution of resistance to the insecticide diazinon in populations of Australian sheep blowfly [52, 53]. Here, an early resistance mutation arose conferring higher fitness in the presence of insecticide, but lower fitness compared to wildtype in the absence of insecticide. A second mutation then evolved to ameliorate the deleterious mutation, thus restoring fitness to wildtype levels for the double mutants.

A key issue in evolutionary biology is understanding the extent to which epistasis, and in particular serial epistasis, determines the path of evolutionary change [51]. Such evolutionary constraints have been shown clearly at the level of individual proteins, for example, in a classic study of the evolution of novel function in vertebrate steroid receptors [54], the authors evaluated experimentally the inferred ancestral proteins leading to the separate evolution of mineralocorticoid and glucocorticoid steroid receptors. They found that structural interactions imposed constraints that determined a specific ordering for the observed evolutionary substitutions. At the same time, the importance of serial epistasis in larger-scale systems such as regulatory networks is less well understood [55]. Our results suggest that whack-a-mole sensitivity will evolve as an emergent property of the network when there is distributed sensitivity and the serial epistasis effects that come with it.

Taken together, under conditions of strong antagonistic coevolution, sensitivity in gene regulatory networks evolves to be broadly distributed and highly labile. Our results suggest there will be no central network elements that determine phenotype changes in the long term. Previous studies had found that network modularity could evolve in the context of alternating environments comparable to those that emerge in our model [22, 23]. A modular network architecture can facilitate phenotype switching by perturbing key interaction(s) between modules [26]. However, we observe an entirely different mechanism based on sensitivity in which modularity does not play a role. When we compared with the Espinosa-Soto model [23], we found that modularity did not evolve even when we adopted many model features, and perhaps the most relevant difference with that model lies in the nature of the perturbations (S11 Fig). Modularity may be more likely to evolve in the face of environmental perturbations than in networks faced predominantly with mutational perturbations [25, 26, 56]. When we compared with the Kashtan model [22], we found that introducing Modularly Varying Goals (MVGs) could, to some extent, drive the evolution of persistent sensitive interactions (S12B Fig), although network modularity did not increase (S12C Fig). As we have observed, distributed sensitivity offers the advantage of allowing a large number of mutations throughout the network to generate phenotype changes. If a network has many different regulatory interactions that enable rapid adaptation via point mutations, the network does not have to mutate a specific interaction back-and-forth in order to repeat the process.

## Methods

### Host-Parasite Coevolution Model

The model largely follows previously published models [34–37] with the exception of selection, which here depends on interactions between the host and parasite populations. In our model, a genotype is represented as a matrix (*W*) where the elements *w*_*ij*_ describe the regulation of the *i*−*th* gene by the *j*−*th* gene product. Positive matrix entries represent activation, negative entries represent repression and zeros indicate no interaction. Gene expression is represented by a vector *S*(*t*) containing elements *S*_*i*_(*t*) representing the expression level, in the range (0,1), over time *t* of the *i*−*th* gene. Initial gene expression, *S*(0), is given as a random binary vector of 0 and 1 expression levels. Gene expression dynamics are determined by the difference equation *S*(*t*+1) = *σ*(*W S*(*t*)) where $\sigma \left(x\right)=\frac{1}{1+e^{-ax}}$ is a sigmoid function. The steady state gene expression,$\;\hat{S},$ is the phenotype and individuals not reaching steady state have zero fitness. The evolutionary simulation is initiated by creating a founder individual for each population in the form of a random matrix *W* of regulatory interactions containing non-zero elements with probability *c*, drawn from a Normal distribution, *N*(0,1). The founder is copied to form the initial population of size *M*. Each population undergoes cycles of reproduction, mutation and selection. In the case of sexual reproduction candidate offspring are produced by inheriting a row in the matrix *W* at random from either parent. Here each row *i* represents regulatory interactions of the set of *cis*-regulatory elements controlling the expression of gene *i*. Row-wise inheritance implies inheritance of *cis*-regulatory regions and free recombination among loci. Under asexual reproduction, random parent genotypes are simply cloned. Following [36], mutations apply to the genotype of each offspring, *W* and may cause addition of new network interactions (when element *w*_*ij*_ = 0) or deletions and modifications (when *w*_*ij*_ ≠ 0). The mutation frequency per genotype is constant (*μ*). Mutations lead to either addition (*ρ*), deletion (*ϕ*) or modification (*δ*) of interactions. The addition and deletion rates are set to ensure that network density does not change from its initial value (See Parameters section below). In the selection step, the interaction between host and parasite populations determines a distinct fitness function for each population, as described in the main text.

### Parameters

Unless otherwise stated, the simulation results used the following parameter values: number of genes, *N* = 10; population size, *M* = 200; mutation rate per genotype, *μ* = 0.1; selection strength, *α* = 0.1; asexual reproduction; network density, *c* = 0.5. As described previously,[36] the network density *c*, will be at steady state when its difference in time, Δ*c*(*t*) = *c*(*t*)−*c*(*t*−1) = *μ*(*α*(1−*c*(*t*))−*ϕc*(*t*))/*N* ^2^ is zero. We therefore chose the parameters for addition (*ρ* = 0.025) and deletion (*ϕ* = 0.025) that satisfy Δ*c*(*t*) = 0. Given these parameters, modifications are set to (*δ* = 1−*ϕ*).

### Sensitivity Score

As described above, a mutation is defined as the replacement of one element *w*_*ij*_ (*i*,*j* = 1,…,*N*) with a random number drawn from a Gaussian distribution, *N*(0,1) if the interaction is either modified or added and with zero if the interaction is deleted. The sensitivity score is calculated by estimating the expectation of a phenotype inversion given a random mutation. This involves evaluating whether a mutation that would change *w*_*ij*_→*l* will generate a phenotype inversion (*k*(*l*) = 1) or not (*k*(*l*) = 0). Because the probability of the mutation *w*_*ij*_→*l* follows a continuous Gaussian distribution *f*(*l*), we employ a discrete approximation given by evaluating *f*(*l*) at 2*L*/*δ*+1 positions across the range [−*L*, *L*] separated by small intervals of size *δ*. More formally, the sensitivity score of an interaction (*w*_*ij*_) in a network is measured as $SS_{ij}=\sum_{l=-L}^{L}\delta \cdot f(l)\cdot k(l)$ where *l*∈{−*L*+*n*⋅*δ* | −L+*n*⋅*δ*≤*L*, *n*∈ℤ*} (= the range of mutation: *w*_*ij*_→*l*),$f(l)=\;\frac{1}{\sigma \sqrt{2\pi }}e^{-\frac{l^{2}}{2\sigma^{2}}}$ (normal distribution probability density function with mean = 0) and *k*(*l*) = 1 if the phenotype is inverted by the perturbation *w*_*ij*_→*l*, otherwise *k*(*l*) = 0. We consider a phenotype as inverted if the *L*_1_ distance ($\|X-Y{\|}_{1}=\sum_{i=1}^{N}\left|x_{i}-y_{i}\right|$) between the original phenotype and a perturbed phenotype by the *w*_*ij*_→*l* mutation, excepting those *N*_*b*_ genes that have basal expression (*s*_*i*_ = 0.5) due to not having inputs, is greater than *p*_*flip*_ (*N*−*N*_b_). For all results reported we used *p*_*flip*_ = 0.9 and *δ* = 0.02, *σ* = 1 and *L* = 3, which covers the range of 99.73% of possible mutations at *w*_*ij*_. The *SS* of a genotype (*W*) is the average of *SS*_*ij*_ for all elements *w*_*ij*_, i.e., $SS=\sum_{i=1}^{N}\sum_{j=1}^{N}\frac{SS_{ij}}{N^{2}}$.

### Lability of Sensitive Interactions

The overlap in sensitivity, which describes the similarity of two genotypes, *u* and *v*, is measured as the Jaccard index, $J\left(u,v\right)\;=\;\frac{\left|A_{u}\cap A_{v}\right|}{\left|A_{u}\cup A_{v}\right|}$, where *A*_*u*_ is the set of sensitive interactions in *u* for which *SS*_*ij*_>0 and *A*_*v*_ is the set for genotype *v*. We calculate *J*(*u*,*v*) for an individual (*u*) and its ancestor (*v*) of the same phenotype. Comparing with an ancestor of the same phenotype is fairer than comparing with an inverted phenotype. Because it is not possible to guarantee that an ancestor at a particular previous generation will have the same phenotype, we chose the closest ancestor having the same phenotype within a window of size 10 (i.e., assuming intervals of size 100, ancestors in ranges of 1–10,11–20,.., 91–100 generations previous). As a null model, sensitive interactions, with *SS*_*ij*_>0 are randomly redistributed in the networks. The overlap in sensitivity for the null model is calculated in the same way and the mean overlap for 100 null models is used (Fig 3A).

### Null Model for Distribution of Sensitivity in Sensitive Interactions

Assuming that a network has *x* interactions with sensitivity score *SS*_*ij*_>0 and the sum of these *x* sensitivity scores is *H*. For the null model we used a standard string cutting method that generates *x* numbers such that their sum equals *H*. This was implemented by choosing *x*−1 random numbers in the range (0,*H*) and then calculating the distances between the adjacent numbers including the end points 0 and *H*. These distances (of which there are *x*), are random numbers which are distributed according to a Dirichlet distribution, and whose sum is *H*. As we did for the original network, we calculated the standard deviation (SD) of these *x* randomly distributed sensitivity scores. We repeated this process 100 times and compared the mean value with the SD of the original network (S5A Fig).

### Measuring Environmental Robustness

To quantify environmental robustness we perturbed initial gene expression 500 times for each individual in the population by changing *s*_*i*_→1−*s*_*i*_ at a rate 0.01/gene (S7A Fig) and 0.2/gene (S7B Fig). We then calculated the phenotype distance $D\left(S_{1},S_{2}\right)=\frac{\sum_{i=1}^{N}\left|S_{1}{}_{i}-S_{2}{}_{i}\right|}{N}$ between unperturbed (*S*_1_) and perturbed (*S*_2_) phenotypes excluding phenotype inversion cases as the measure of environmental robustness.

### Evolution of Modularity under Coevolutionary Selection

The modularity measure we used is taken from [57]. We restate the definition as follows: Given a graph *G*(*V*,*E*), where |*V*| = *k*,|*E*| = *l*, the vertices of *G*(*V*,*E*) can be clustered into *n* clusters, *C* = {*C*_1_, *C*_2_, …, *C*_*n*_}, 1≤*n*≤*k*. Modularity is defined as $Q\left(C\right)=\sum_{i=1}^{k}\left[\frac{\left|E\left(C_{i}\right)\right|}{l}-{\left(\frac{\sum_{v\in C_{i}}\text{deg}\left(v\right)}{2l}\right)}^{2}\right]$ where *E*(*C*_*i*_) = {{*v*_*h*_,*v*_*t*_}∈*E*|*v*_*h*_,*v*_*t*_∈*C*_*i*_} and deg(*v*) = |{∀*v*_*t*_≠*v*|{*v*,*v*_*t*_}∈*E*}|.

We adapted the (Espinosa-Soto) model described in [23] to our antagonistic coevolution model as follows. The previous study used two target phenotypes simultaneously, each of which has a conserved part and a distinct part as input gene expressions. To emulate this in our model, we assigned half the genes to be under stabilizing selection and the other half under coevolutionary selection. The set of genes under stabilizing selection has the same target phenotype throughout the simulation whereas the other is under coevolutionary selection as with the model described in the main text. To represent environmental perturbations, the input gene expression is perturbed by changing *s_i_*→1−*s*_*i*_ at a rate 0.15/*N* as described in [23], for 400 experiments. The fitness function of an individual is *f* = 1−*e* ^−3γ^, $\gamma =\sum_{i=1}^{400}{\left(1-D_{i}/D_{max}\right)}^{5}/400$, where *D*_*i*_ is the Hamming distance between the target phenotype and a new phenotype from *i th* perturbed input. For the model version that assigns half of the genes to be under stabilizing selection and the other half to be under coevolutionary selection but does not include environmental perturbations, we calculate two types of fitness for stabilizing and coevolutionary selection respectively:

$$f_{s}=\;1-e^{-\frac{d}{\alpha }},\;d=\overset{N_{s}}{\underset{i=1}{\sum }}{\left(S_{optimal}\left(i\right)-\hat{S}\left(i\right)\right)}^{2}/\left(N_{s}\cdot \zeta \right)$$

and

$$f_{c}=\{\begin{array}{c} e^{-\frac{1-d}{\alpha }},\;\;\;host\\ e^{-\frac{d}{\alpha }},\;\;parasite \end{array},\;d=\overset{N_{c}}{\underset{i=1}{\sum }}{\left(S_{antagonist}\left(i\right)-\hat{S}\left(i\right)\right)}^{2}/\left(N_{c}\cdot \zeta \right).$$

*ζ* = 1 for continuous expression levels and *ζ* = 4 for discrete (-1,+1) expression levels. *N*_*s*_ is the number of genes under stabilizing selection and *N*_*c*_ is the number of genes under coevolutionary selection. Survival requires both *f*_*s*_ and *f*_*c*_ to exceed a uniformly-distributed random value in the range [0,1].

## Supporting Information

S1 FigFitness function for host (red) and parasite (blue) for different selection pressure strengths (*α*).
$D\left(S_{1},S_{2}\right)=\frac{\sum_{i=1}^{N}{\left(S_{1}{}_{i}-S_{2}{}_{i}\right)}^{2}}{N}$ is the distance between two phenotypes *S*_1_ and *S*_2_. Host and parasite fitness values are symmetric about *D* = 0.5.(TIFF)

S2 Fig Host and parasite populations used to generate Fig 1B. Here, as in Fig 1B, genes are shown on the horizontal axis and evolutionary time in generations on the vertical axis. The colors represent the gene expression levels of every gene in every individual, as indicated in the color bar.
(TIFF)

S3 Fig Effect of parameter changes on the evolution of sensitivity and robustness. Results analogous to Fig 2 (for sensitivity and robustness) for varying parameters of the model. In each case, we only vary one parameter, maintaining the others fixed. (A) and (B) are for different values of the selection strength, *α* (*c* = 0.3,*M* = 200,*μ* = 0.1); (C) and (D) are for population size, *M* (*c* = 0.3,*σ* = 0.1,*μ* = 0.1); (E) and (F) for network density, *c* (*σ* = 0.1,*M* = 200,*μ* = 0.1); (G) and (H) are for mutation rate, *μ* (*c* = 0.5,*σ* = 0.1,*M* = 200).
(TIFF)

S4 Fig Analysis of cases with multiple mutations. In the case of (A) a single typical simulation, and (B) averaged over 100 simulations, we compared the genotype of each individual with the ancestor genotype by back-tracking asexually reproducing populations. Curves show the frequency of single (red) and multiple (2 (green), 3 (blue), and 4 (cyan)) mutations over time. Error bars represent one SEM. (C) For the same simulation shown in (A), we measured the sensitivity score at those interactions that mutated when there were two mutations. The higher of the two sensitivity scores is shown in red, and the lower of the two is shown in blue. The error bars represent one SD.
(TIFF)

S5 Fig Emergence of sensitive interactions and distribution of sensitivity score (*SS*) among the sensitive interactions. (A) Standard deviation (SD) of sensitivity scores at sensitive interactions for which *SS*_*ij*_>0, in red for host, blue for parasite. Null model results (see Methods) are also shown for host in magenta and for the parasite in cyan. The observed SD is comparable and even slightly below the SD of the null model. (B) As coevolution proceeds, the fraction of sensitive interactions in the network for which *SS*_*ij*_>0 increases monotonically reaching a plateau in both host (red curve) and parasite (blue curve).
(TIFF)

S6 Fig Evolution of sensitivity and robustness under sexual reproduction. The results for sexual reproduction are qualitatively equivalent to those for asexual reproduction. However, for many parameter combinations in which robustness evolves under asexual reproduction, it does not evolve to be higher than the initial (random) case under sexual reproduction. Here, in plot (B) we show an example (parameters: *c* = 0.7,*M* = 500,*α* = 0.1) for which the robustness clearly evolves.
(TIFF)

S7 Fig Progression over time of phenotype distance in response to perturbations of the initial conditions to evaluate environmental robustness. The initial gene expression levels were perturbed 500 times for each individual in the population (A) at a rate 0.01/gene and (B) 0.2/gene. The phenotype distance was used (see Methods) to evaluate the environmental robustness.
(TIFF)

S8 Fig (A) Sensitivity and (B) robustness over time for asymmetric population sizes. We tested one order of magnitude difference between host population size = 100 and parasite population size = 1000. These plots are in the same format as for Fig 2; all other parameter values are the same as for Fig 2.
(TIFF)

S9 Fig Distribution of higher-level (row) sensitivity in the population. A) Every 50 generations we determined the sum of sensitivity scores (*SS*_*ij*_) for each row in every individual in the host population of a typical simulation. In particular, we tracked the row *i*_*max*_ for which the value of *SS*_*i*_ is maximal within each individual. The row *i*_*max*_ of each individual is indicated by a different color, and the profile of for the entire population is represented by a row. We sampled these values every 50 generations (vertical axis). Light blue was used (NA on color bar) if there are no sensitive interactions in that particular network. For example, in generation 2000, shown at the bottom of the plot, 53 out of 200 individuals had *i*_*max*_ = 2. We define a “dominant” row as existing when more than half of the population has the same *i*_*max*_. Thus for example, at generation 500, row *i*_*max*_ = 8 is dominant because more than half the individuals in the population have *i*_*max*_ = 8 (light purple). Row 10 (beige) on the other hand, is never dominant. The green curve in plot (B) shows, in rank order, the frequencies with which *i*_*max*_ was dominant for each generation in plot (A). In most cases there was no dominant row and we classified these cases as “row 0”. For this analysis we considered only populations in steady state, i.e. from generation 500 onwards. For example, in plot (A), row 4 and 8 were dominant in 5.56% of the generations for each, more than any other row (rank #1), and this is shown in plot (B) as green dots at (1,0.0556) and (2,0.0556). The red curve shows the mean values for 100 independent simulations.
(TIFF)

S10 Fig Evolution of diversity with an initial phase of stabilizing selection. As described in the main text, we measure diversity in the population as the fraction of distinct networks in the population (simplified to sign form: +1/0/-1). We performed simulations with an initial phase of 500 generations under stabilizing selection (magenta and cyan curves) and include here the original results without the initial phase for comparative purposes.
(TIFF)

S11 Fig Evolution of modularity in different model variants. A) The table describes features (1^st^ column) that are different between our model (M1, described in 2^nd^ column) and the Espinosa-Soto model (M2, described in 3^rd^ column). Using the measure defined in Espinosa-Soto and Wagner, we measured modularity in our model (curve C1 in plot) and reproduced the results of model M2 (curve C4). We further tested two variant models that had features of both models, as indicated in columns 5 and 6, which correspond to curves C2 and C3 in the plot. The variant models did not show increased modularity over time. In these simulations, coevolution begins at generation 500 for all models C1 ~ C4. Initial network density, *c* = 0.3 for all four models to match the parameters used in Espinosa-Soto and Wagner. To make the models comparable, for models C2 and C3, we adopted the convention in the M1 model of defining only half the genes using the opposite population (either host or parasite) as a reference phenotype for defining fitness. The remaining genes used the founder individual, as in the stabilizing selection model without antagonistic coevolution, as in initial phase of S10 Fig.
(TIFF)

S12 Fig Persistent/dominant sensitive interactions appear under selection for repeatedly switching Modularly Varying Goals (MVGs). (A) Fitness vs time for MVGs that switch every 50 generations. Fitness drops when the goal is changed and reaches equilibrium within approximately 20 generations. (B) Distribution of the frequency of being a sensitive interaction among all ***N***×***N*** interactions. This figure is the equivalent of Fig 4C for the case of MVGs. The bottom figure presents the same data, but has been zoomed in by omitting the left-most data point (fraction = 0). The non-zero tail, and especially those interactions that have frequency of being sensitive = 1, shows there are persistent sensitive interactions. (C) While persistent sensitive interactions do appear under the MVG model as shown in (B), modularity does not evolve because labile sensitive interactions are still present in these networks.
(TIFF)

S13 Fig Distribution of the frequency of interactions being sensitive among all *N*×*N* interactions for a single population under alternating selection strategies. The format is equivalent to Fig 4C. As explained in the main text, in (A) we used two alternating targets in which half of the target genes (N/2) are kept the same as the founder phenotype and the other half are inverted. In (B) we simply alternated between the founder phenotype and its inverted form. In both cases, switching between the two target goals occurs every 50 generations.
(TIFF)

S14 Fig Emergence of sensitivity (A, C) and the distribution of the frequency of interactions being sensitive among all *N*×*N* interactions (B, D) for different addition (*ρ*) and deletion (*ϕ*) rates. The format is equivalent to Figures Figs 2A and 4C respectively (where *ρ* = ***ϕ*** = 0.025). (A) and (B) are results for a 2.5X higher addition/deletion rate of *ρ* = ***ϕ*** = 0.0625, whereas (C) and (D) are for the 2.5X lower addition/deletion rate of *ρ* = ***ϕ*** = 0.01. Other parameters remain as described in Methods (section “Parameters”).
(TIFF)
