# Supplementary figures and images for: Antagonistic Coevolution Drives Whack-a-Mole Sensitivity in Gene Regulatory Networks
Source: PLoS Comput Biol. 2015 Oct 9;11(10):e1004432. doi: 10.1371/journal.pcbi.1004432 (PMC4599961; doi:10.1371/journal.pcbi.1004432)

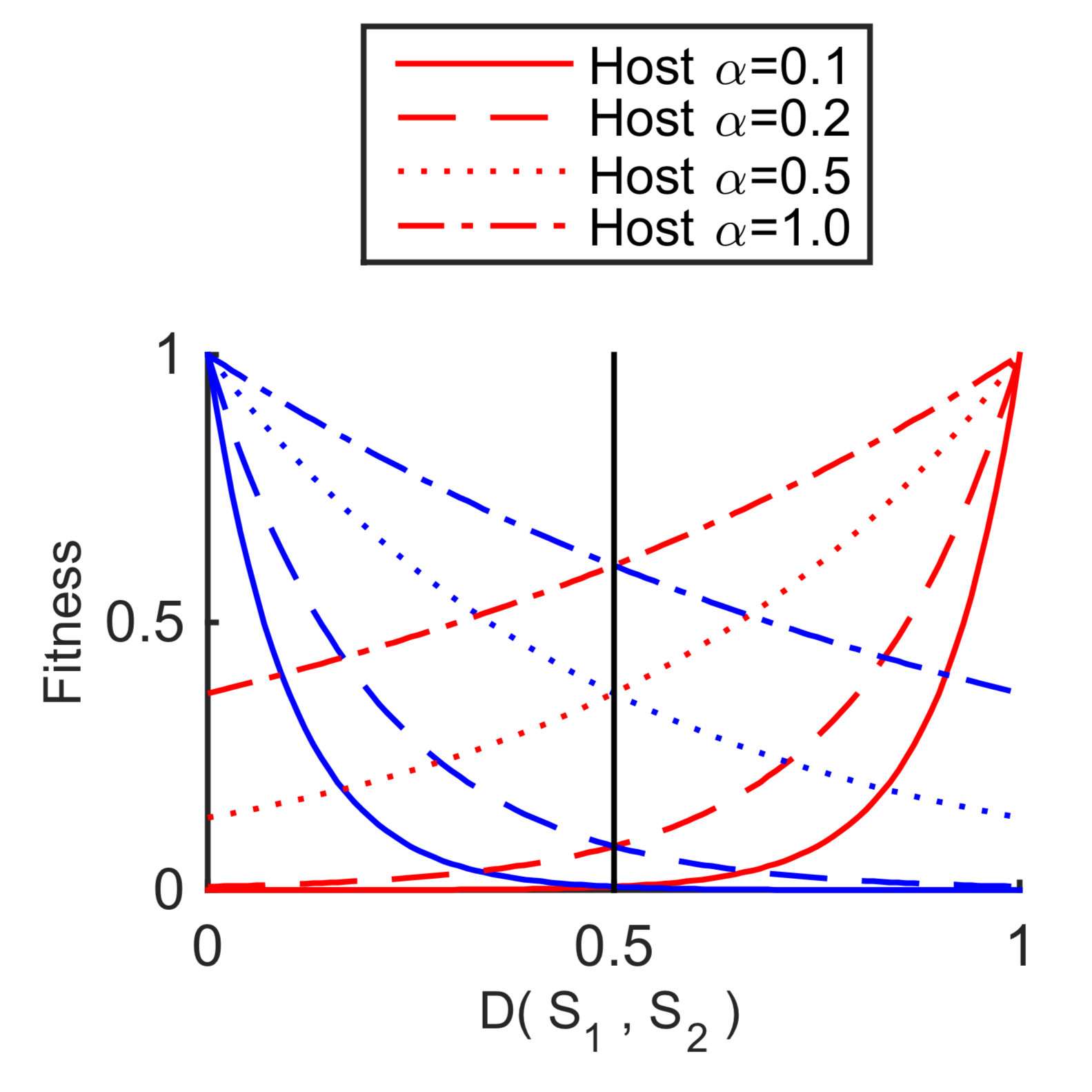

Supplement: S1 Fig — D(S1,S2)=∑i=1N(S1i−S2i)2N is the distance between two phenotypes S 1 and S 2. Host and parasite fitness values are symmetric about D = 0.5. (TIFF) [file pcbi.1004432.s001.tiff]

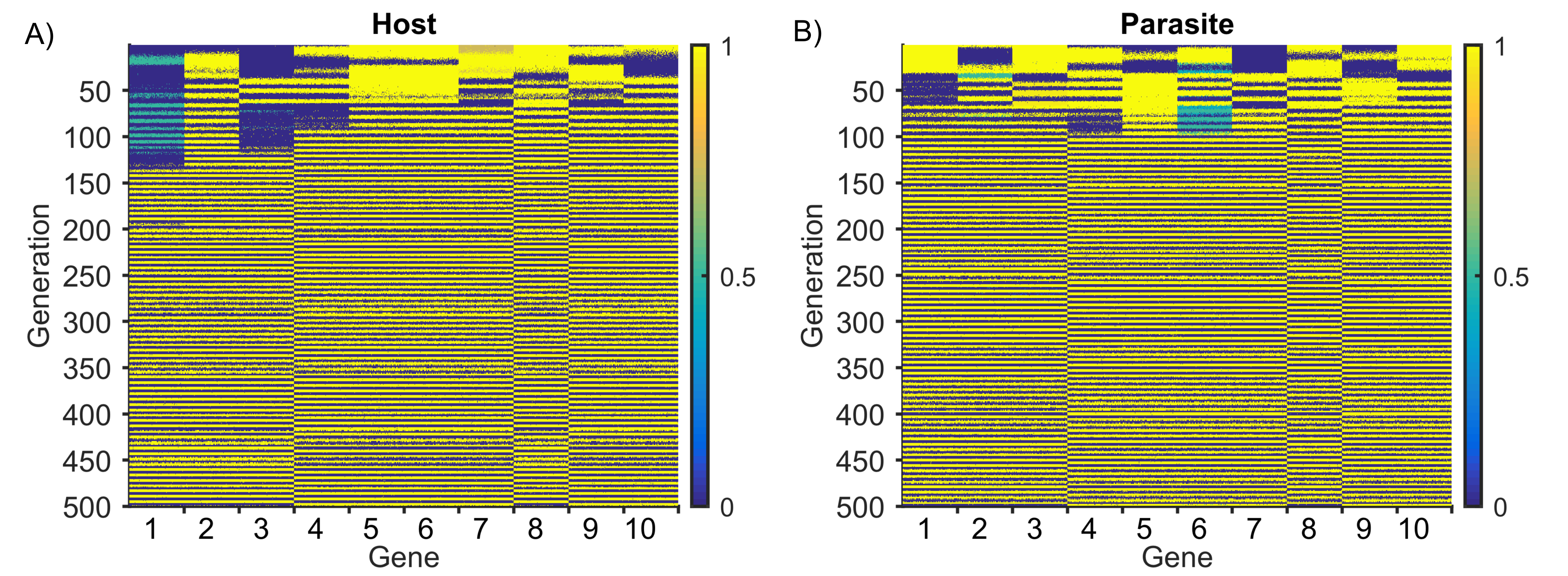

Supplement: S2 Fig — Here, as in Fig 1B, genes are shown on the horizontal axis and evolutionary time in generations on the vertical axis. The colors represent the gene expression levels of every gene in every individual, as indicated in the color bar. (TIFF) [file pcbi.1004432.s002.tiff]

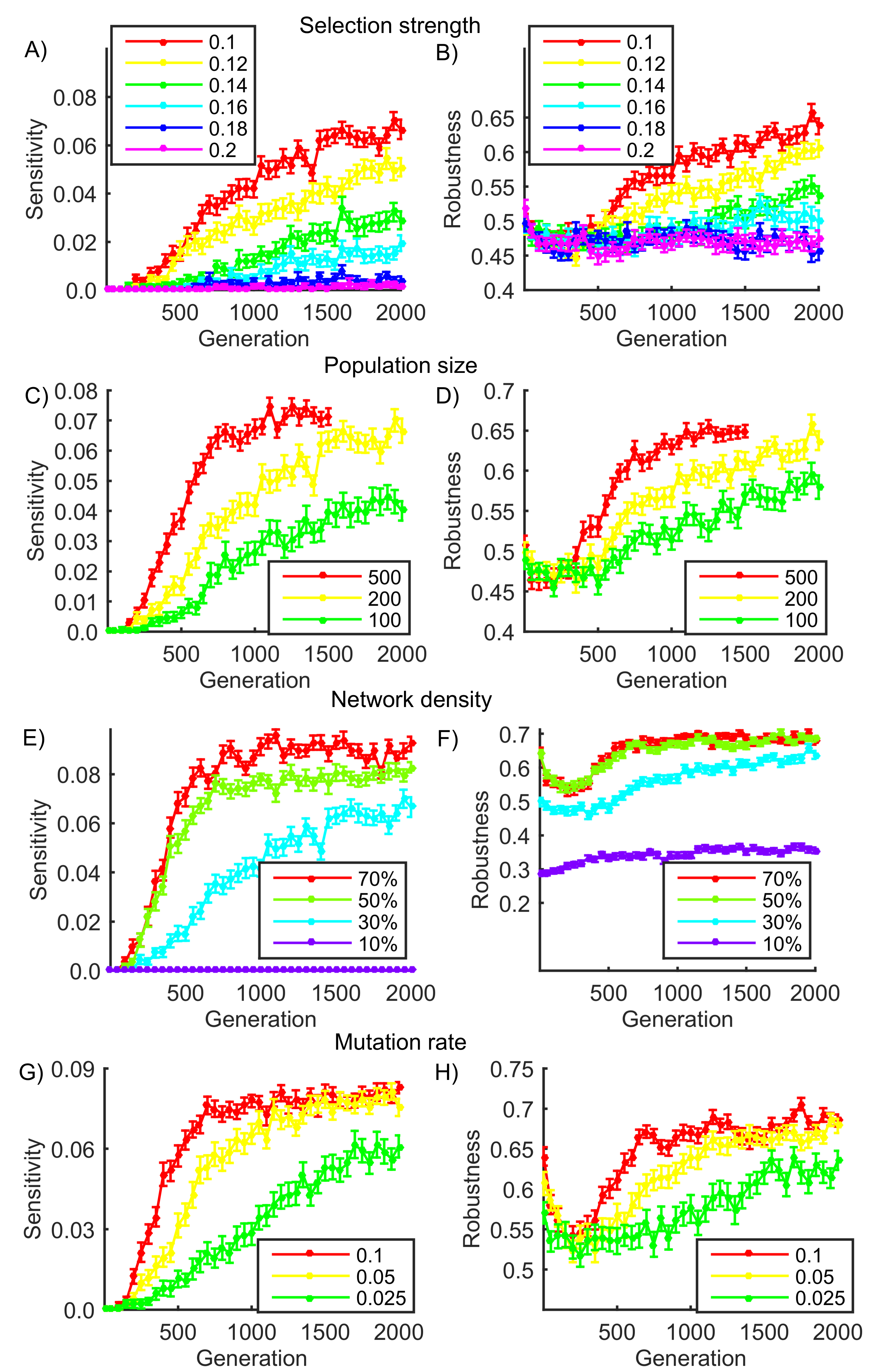

Supplement: S3 Fig — Results analogous to Fig 2 (for sensitivity and robustness) for varying parameters of the model. In each case, we only vary one parameter, maintaining the others fixed. (A) and (B) are for different values of the selection strength, α (c = 0.3,M = 200,μ = 0.1); (C) and (D) are for population size, M (c = 0.3,σ = 0.1,μ = 0.1); (E) and (F) for network density, c (σ = 0.1,M = 200,μ = 0.1); (G) and (H) are for mutation rate, μ (c = 0.5,σ = 0.1,M = 200). (TIFF) [file pcbi.1004432.s003.tiff]

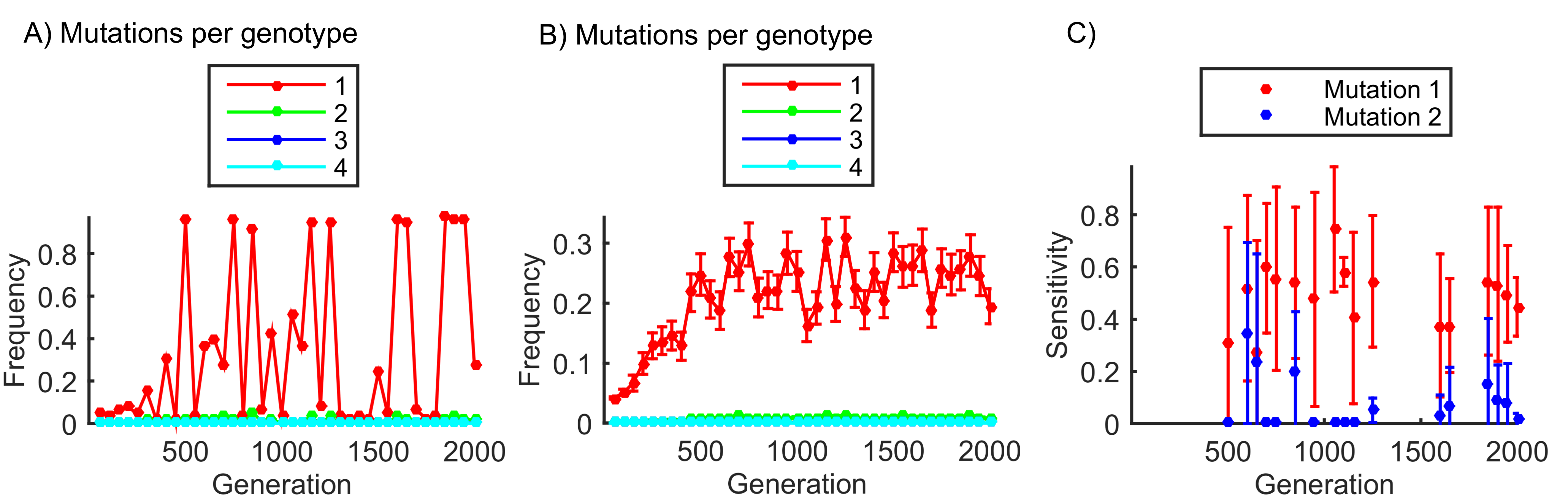

Supplement: S4 Fig — In the case of (A) a single typical simulation, and (B) averaged over 100 simulations, we compared the genotype of each individual with the ancestor genotype by back-tracking asexually reproducing populations. Curves show the frequency of single (red) and multiple (2 (green), 3 (blue), and 4 (cyan)) mutations over time. Error bars represent one SEM. (C) For the same simulation shown in (A), we measured the sensitivity score at those interactions that mutated when there were two mutations. The higher of the two sensitivity scores is shown in red, and the lower of the two is shown in blue. The error bars represent one SD. (TIFF) [file pcbi.1004432.s004.tiff]

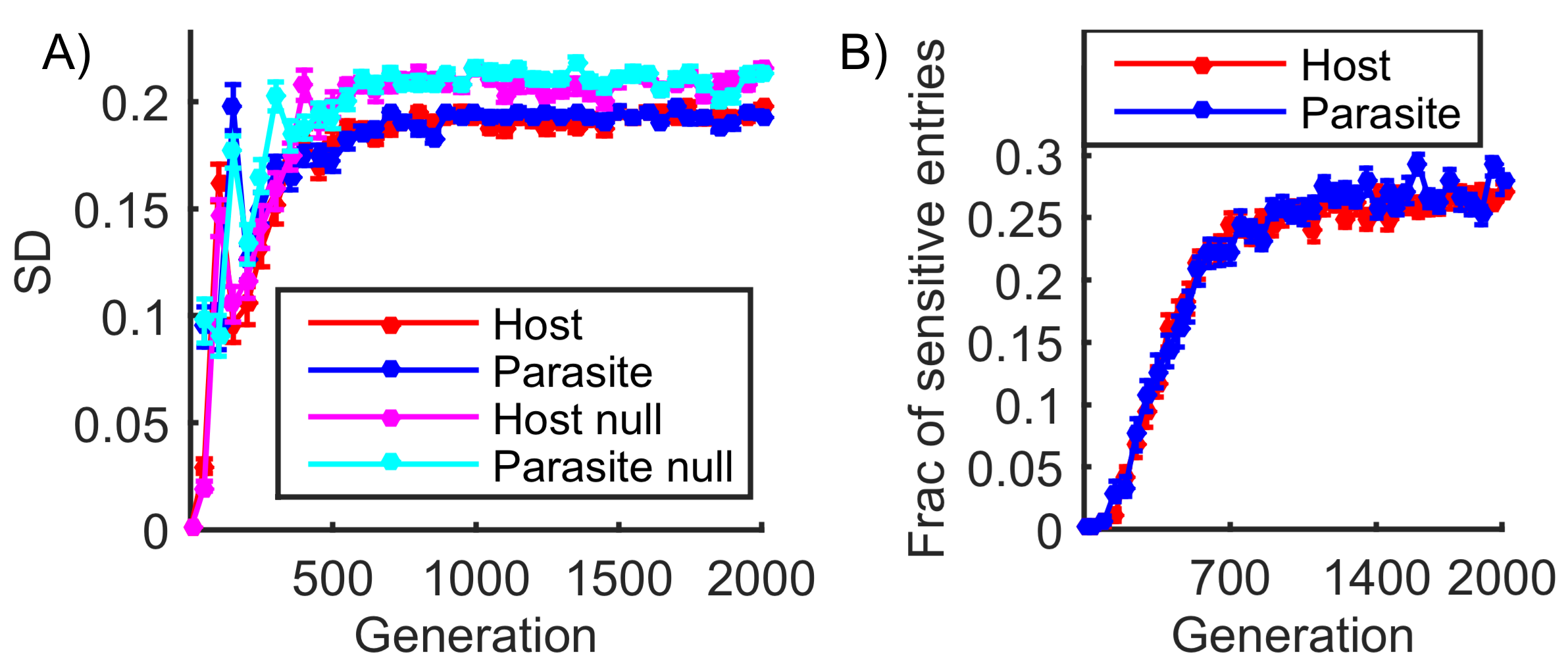

Supplement: S5 Fig — (A) Standard deviation (SD) of sensitivity scores at sensitive interactions for which SS ij>0, in red for host, blue for parasite. Null model results (see Methods) are also shown for host in magenta and for the parasite in cyan. The observed SD is comparable and even slightly below the SD of the null model. (B) As coevolution proceeds, the fraction of sensitive interactions in the network for which SS ij>0 increases monotonically reaching a plateau in both host (red curve) and parasite (blue curve). (TIFF) [file pcbi.1004432.s005.tiff]

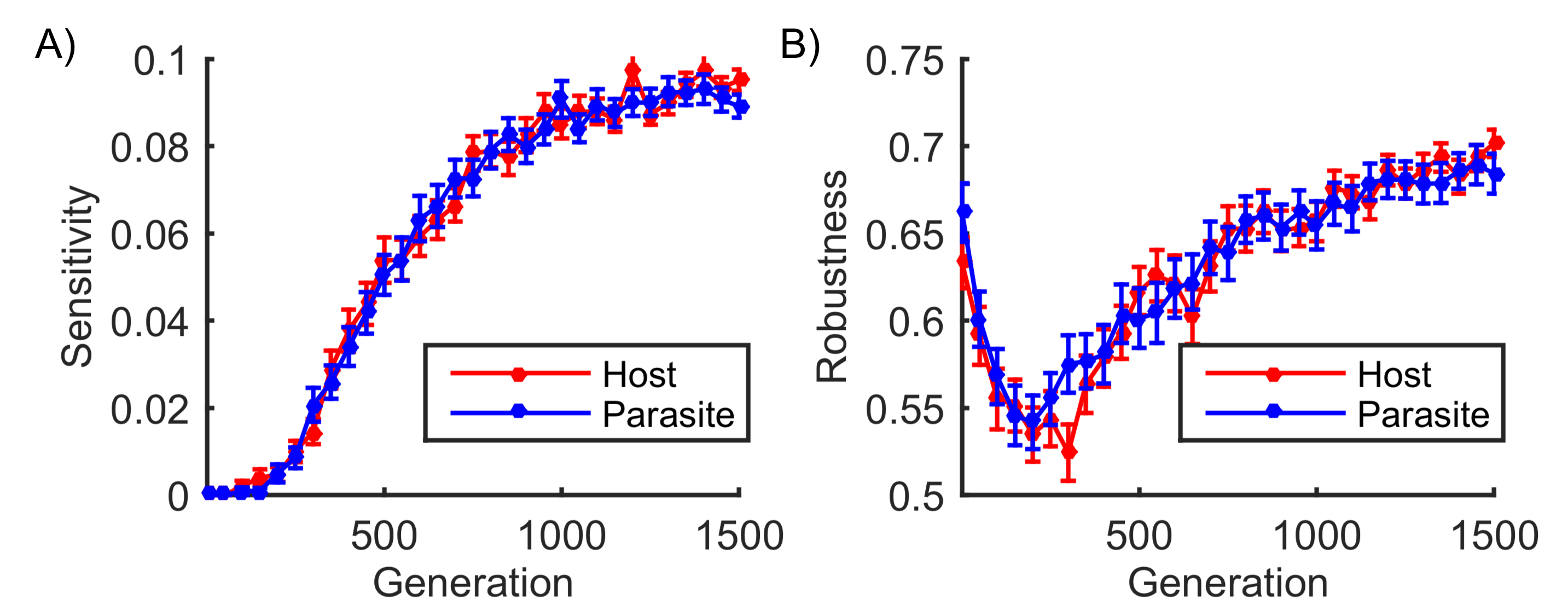

Supplement: S6 Fig — The results for sexual reproduction are qualitatively equivalent to those for asexual reproduction. However, for many parameter combinations in which robustness evolves under asexual reproduction, it does not evolve to be higher than the initial (random) case under sexual reproduction. Here, in plot (B) we show an example (parameters: c = 0.7,M = 500,α = 0.1) for which the robustness clearly evolves. (TIFF) [file pcbi.1004432.s006.tiff]

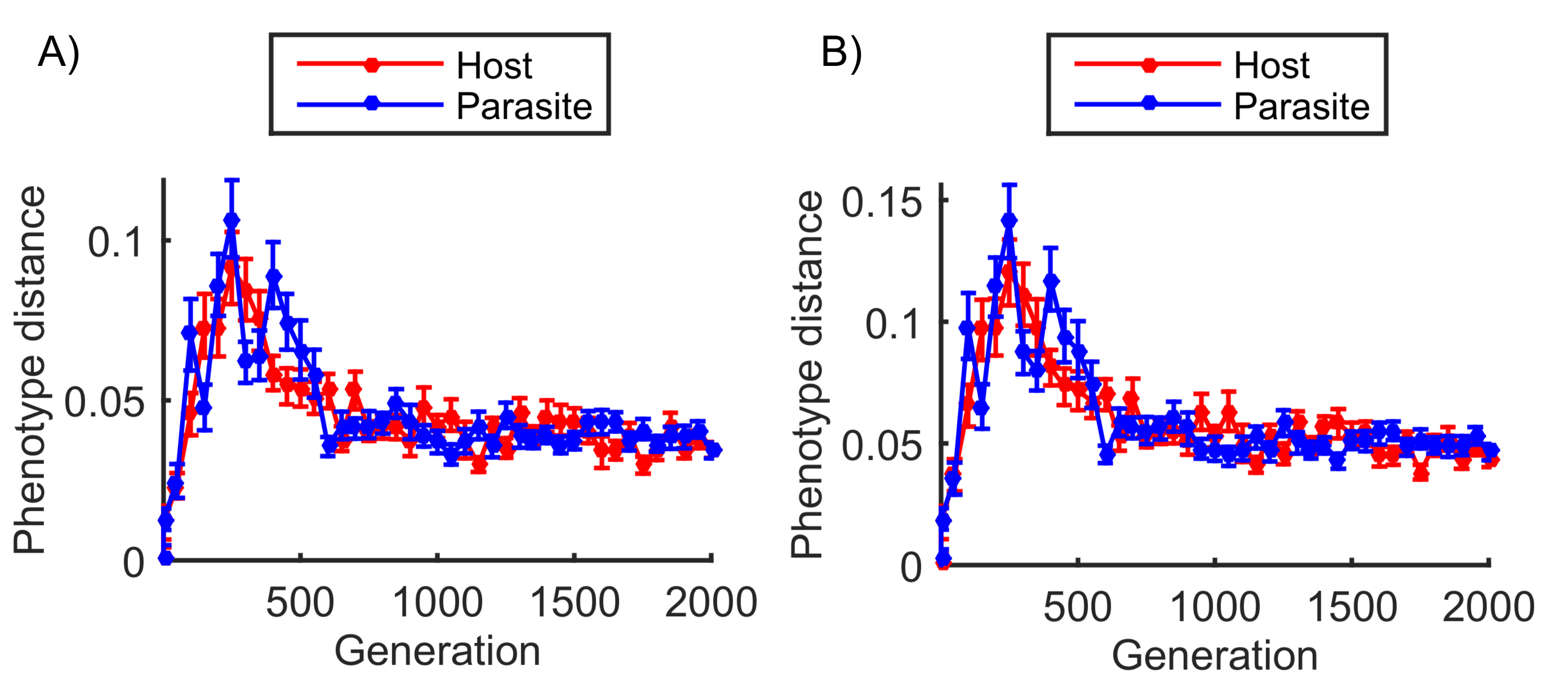

Supplement: S7 Fig — The initial gene expression levels were perturbed 500 times for each individual in the population (A) at a rate 0.01/gene and (B) 0.2/gene. The phenotype distance was used (see Methods) to evaluate the environmental robustness. (TIFF) [file pcbi.1004432.s007.tiff]

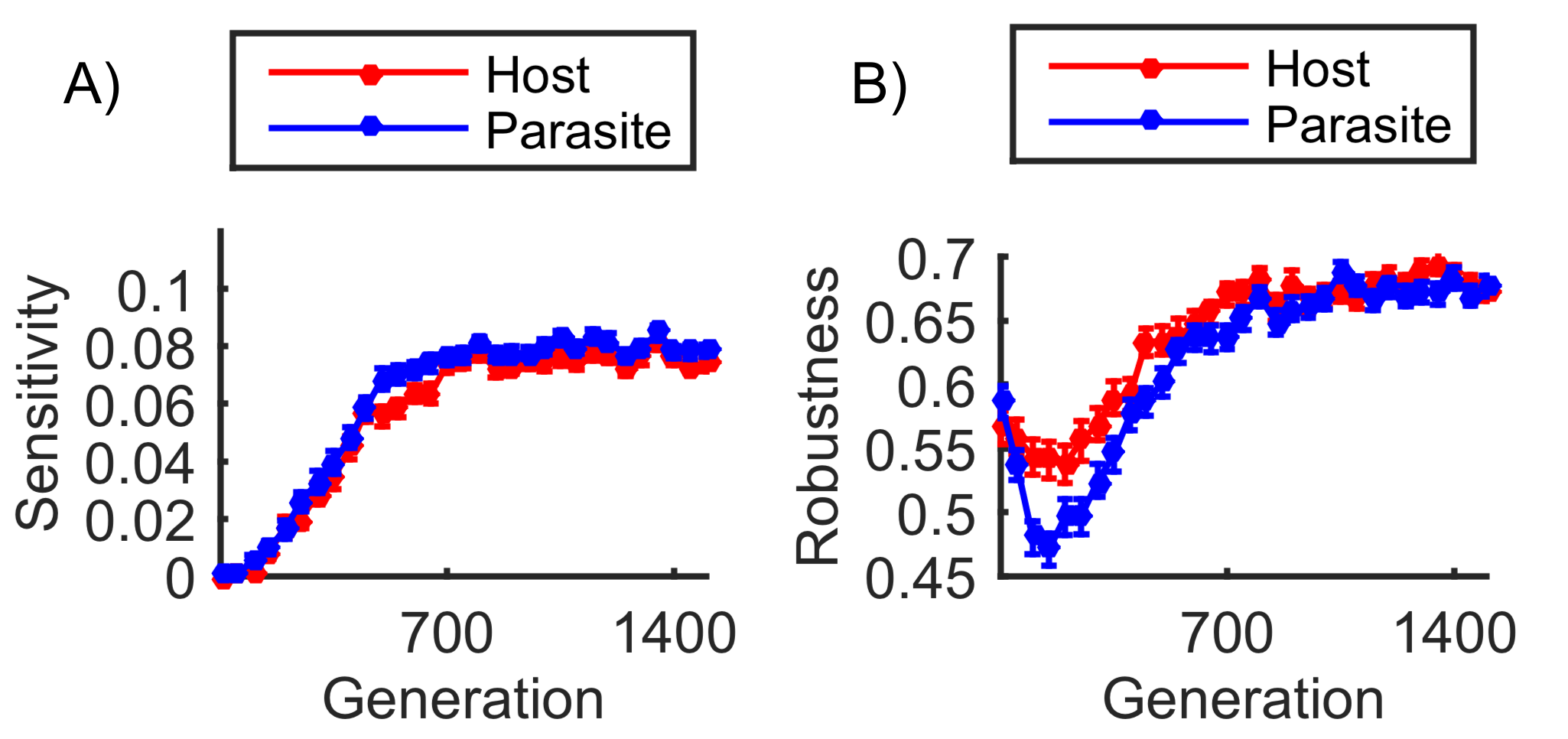

Supplement: S8 Fig — We tested one order of magnitude difference between host population size = 100 and parasite population size = 1000. These plots are in the same format as for Fig 2; all other parameter values are the same as for Fig 2. (TIFF) [file pcbi.1004432.s008.tiff]

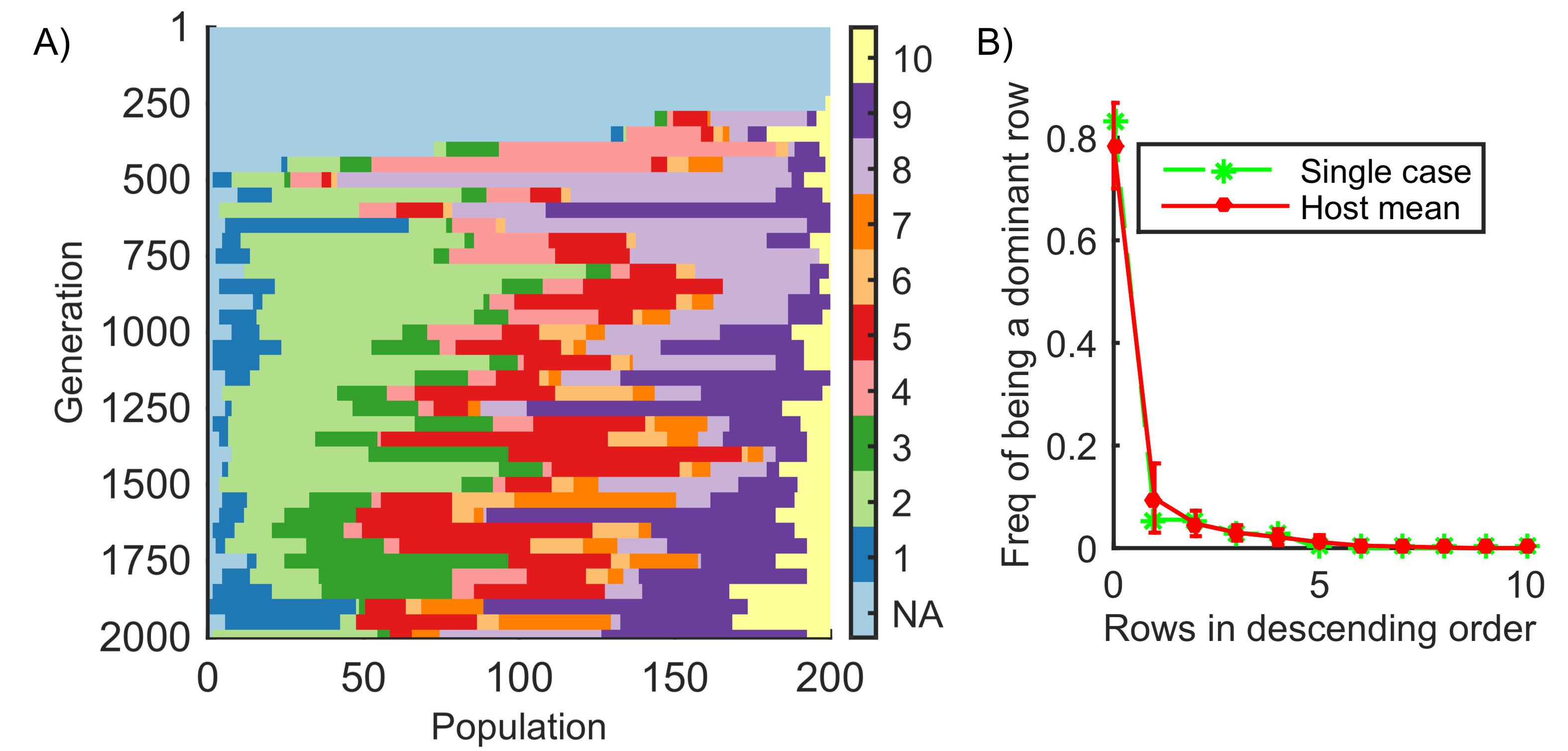

Supplement: S9 Fig — A) Every 50 generations we determined the sum of sensitivity scores (SS ij) for each row in every individual in the host population of a typical simulation. In particular, we tracked the row i max for which the value of SS i is maximal within each individual. The row i max of each individual is indicated by a different color, and the profile of for the entire population is represented by a row. We sampled these values every 50 generations (vertical axis). Light blue was used (NA on color bar) if there are no sensitive interactions in that particular network. For example, in generation 2000, shown at the bottom of the plot, 53 out of 200 individuals had i max = 2. We define a “dominant” row as existing when more than half of the population has the same i max. Thus for example, at generation 500, row i max = 8 is dominant because more than half the individuals in the population have i max = 8 (light purple). Row 10 (beige) on the other hand, is never dominant. The green curve in plot (B) shows, in rank order, the frequencies with which i max was dominant for each generation in plot (A). In most cases there was no dominant row and we classified these cases as “row 0”. For this analysis we considered only populations in steady state, i.e. from generation 500 onwards. For example, in plot (A), row 4 and 8 were dominant in 5.56% of the generations for each, more than any other row (rank #1), and this is shown in plot (B) as green dots at (1,0.0556) and (2,0.0556). The red curve shows the mean values for 100 independent simulations. (TIFF) [file pcbi.1004432.s009.tiff]

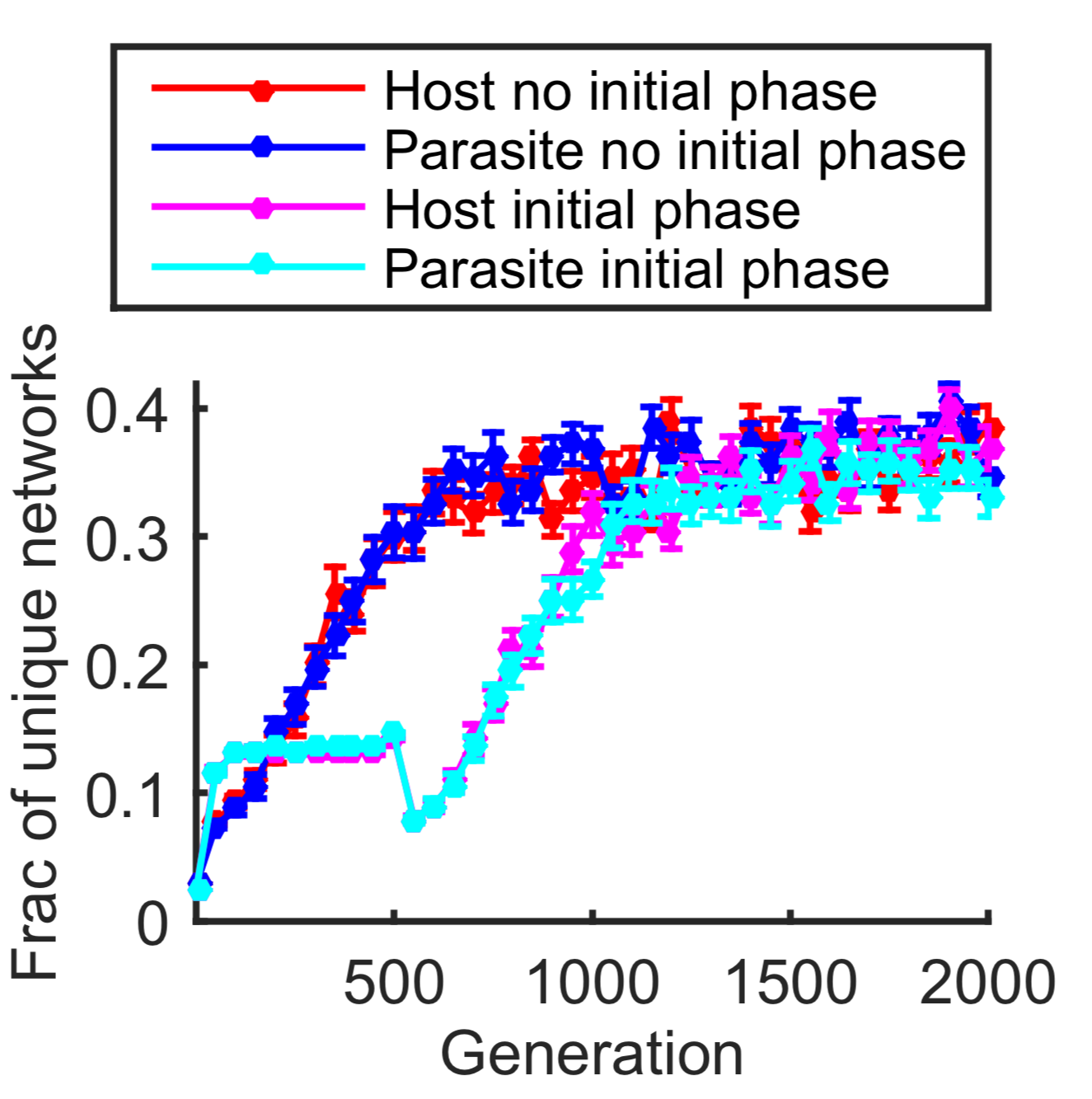

Supplement: S10 Fig — As described in the main text, we measure diversity in the population as the fraction of distinct networks in the population (simplified to sign form: +1/0/-1). We performed simulations with an initial phase of 500 generations under stabilizing selection (magenta and cyan curves) and include here the original results without the initial phase for comparative purposes. (TIFF) [file pcbi.1004432.s010.tiff]

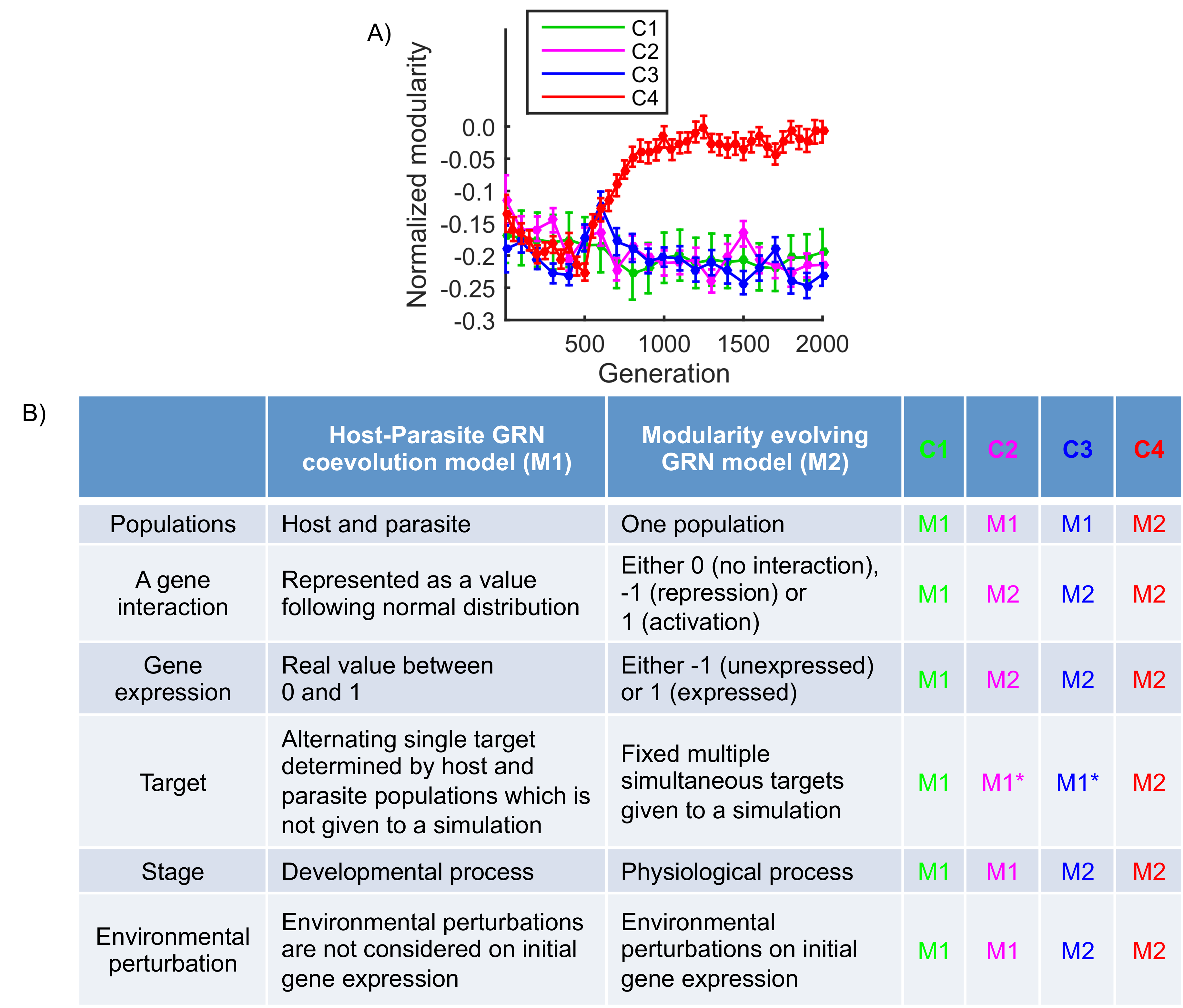

Supplement: S11 Fig — A) The table describes features (1st column) that are different between our model (M1, described in 2nd column) and the Espinosa-Soto model (M2, described in 3rd column). Using the measure defined in Espinosa-Soto and Wagner, we measured modularity in our model (curve C1 in plot) and reproduced the results of model M2 (curve C4). We further tested two variant models that had features of both models, as indicated in columns 5 and 6, which correspond to curves C2 and C3 in the plot. The variant models did not show increased modularity over time. In these simulations, coevolution begins at generation 500 for all models C1 ~ C4. Initial network density, c = 0.3 for all four models to match the parameters used in Espinosa-Soto and Wagner. To make the models comparable, for models C2 and C3, we adopted the convention in the M1 model of defining only half the genes using the opposite population (either host or parasite) as a reference phenotype for defining fitness. The remaining genes used the founder individual, as in the stabilizing selection model without antagonistic coevolution, as in initial phase of S10 Fig. (TIFF) [file pcbi.1004432.s011.tiff]

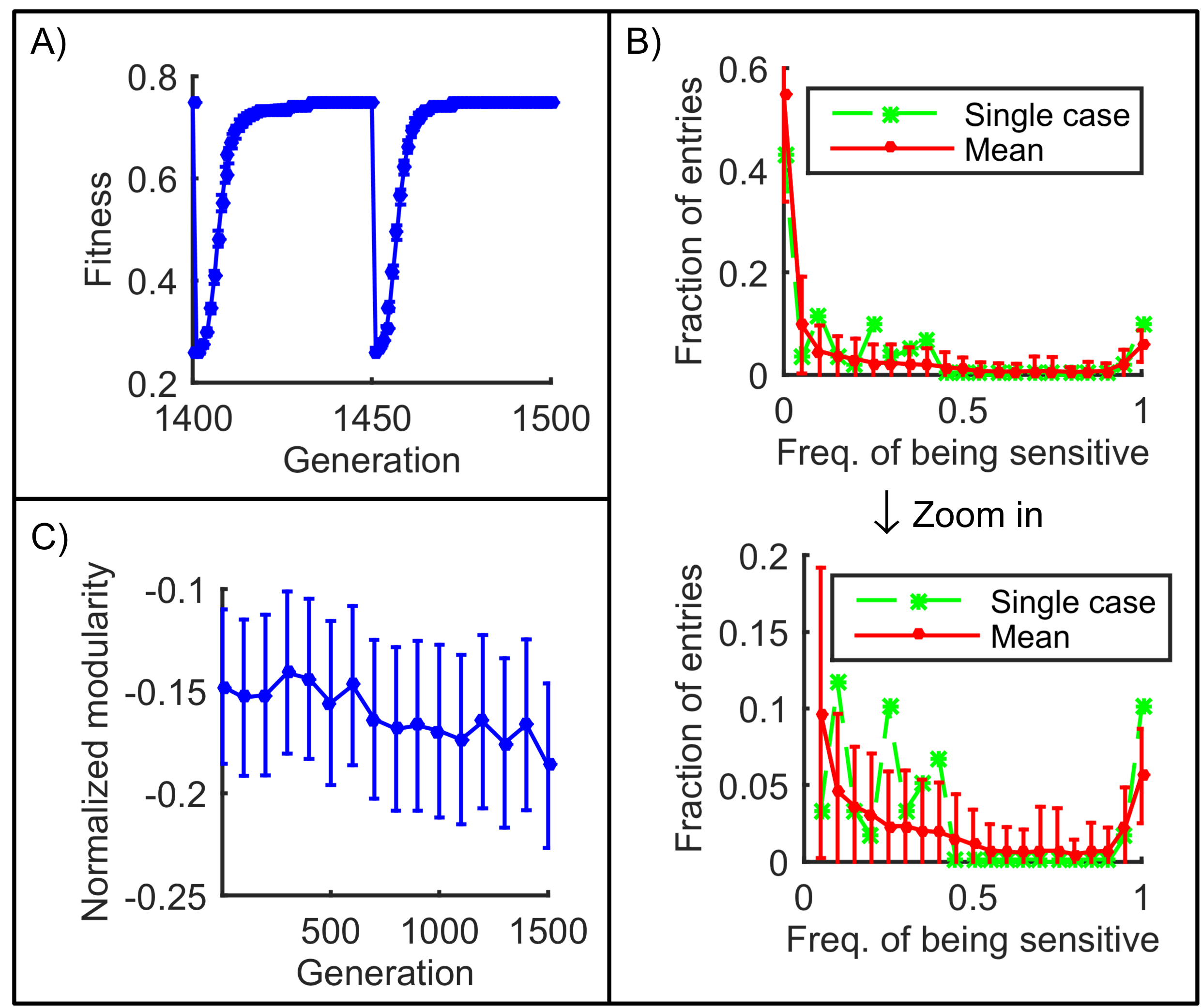

Supplement: S12 Fig — (A) Fitness vs time for MVGs that switch every 50 generations. Fitness drops when the goal is changed and reaches equilibrium within approximately 20 generations. (B) Distribution of the frequency of being a sensitive interaction among all N×N interactions. This figure is the equivalent of Fig 4C for the case of MVGs. The bottom figure presents the same data, but has been zoomed in by omitting the left-most data point (fraction = 0). The non-zero tail, and especially those interactions that have frequency of being sensitive = 1, shows there are persistent sensitive interactions. (C) While persistent sensitive interactions do appear under the MVG model as shown in (B), modularity does not evolve because labile sensitive interactions are still present in these networks. (TIFF) [file pcbi.1004432.s012.tiff]

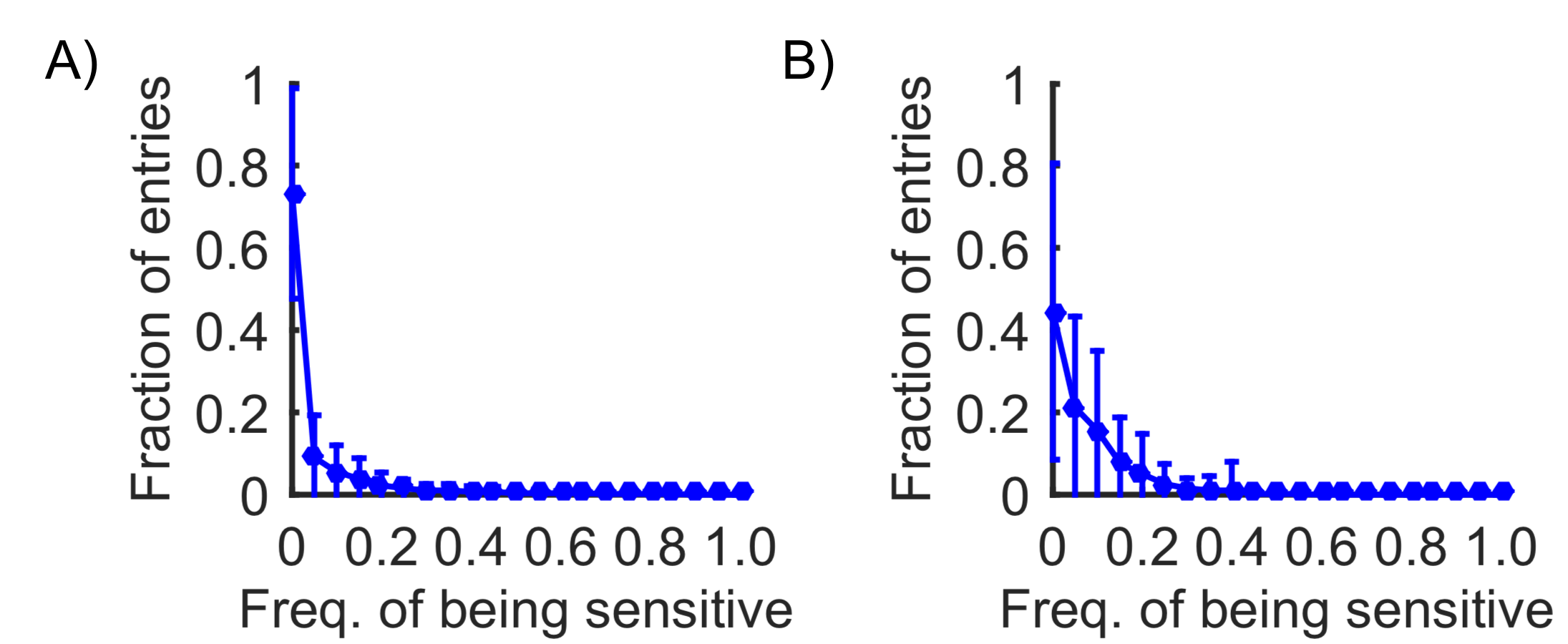

Supplement: S13 Fig — The format is equivalent to Fig 4C. As explained in the main text, in (A) we used two alternating targets in which half of the target genes (N/2) are kept the same as the founder phenotype and the other half are inverted. In (B) we simply alternated between the founder phenotype and its inverted form. In both cases, switching between the two target goals occurs every 50 generations. (TIFF) [file pcbi.1004432.s013.tiff]

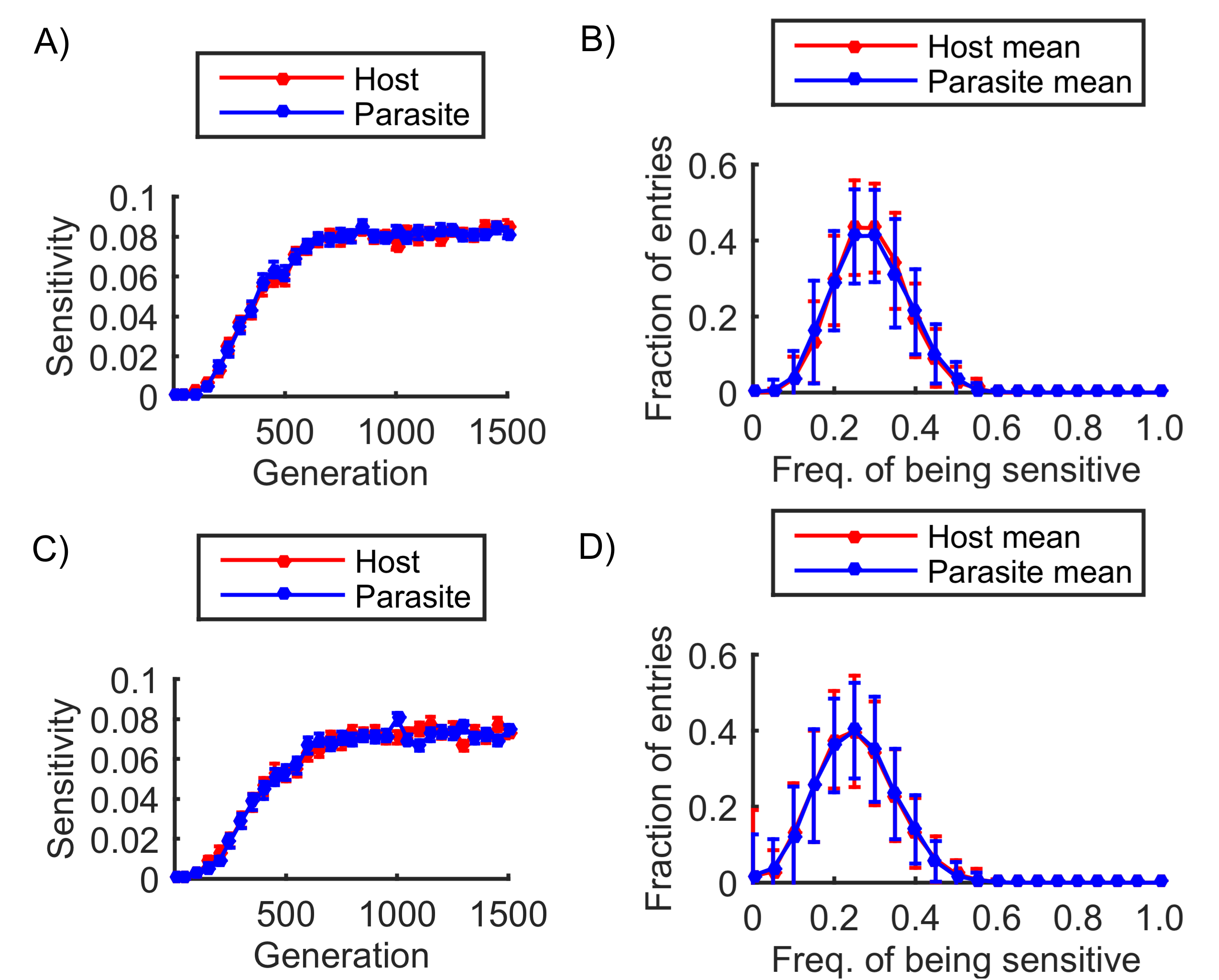

Supplement: S14 Fig — The format is equivalent to Figures Figs 2A and 4C respectively (where ρ = ϕ = 0.025). (A) and (B) are results for a 2.5X higher addition/deletion rate of ρ = ϕ = 0.0625, whereas (C) and (D) are for the 2.5X lower addition/deletion rate of ρ = ϕ = 0.01. Other parameters remain as described in Methods (section “Parameters”). (TIFF) [file pcbi.1004432.s014.tiff]
